# Clinic-Based Mobile Health Decision Support to Enhance Adult Epilepsy Self-Management: An Intervention Mapping Approach

**DOI:** 10.3389/fpubh.2017.00256

**Published:** 2017-10-03

**Authors:** Ross Shegog, Charles E. Begley

**Affiliations:** ^1^School of Public Health, University of Texas Health Science Center, Houston, TX, United States

**Keywords:** epilepsy, self-management, decision support, intervention mapping, intervention, mobile health, electronic health, treatment

## Abstract

**Introduction:**

Epilepsy is a neurological disorder involving recurrent seizures. It affects approximately 5 million people in the U.S. To optimize their quality of life people with epilepsy are encouraged to engage in self-management (S-M) behaviors. These include managing their treatment (e.g., adhering to anti-seizure medication and clinical visit schedules), managing their seizures (e.g., responding to seizure episodes), managing their safety (e.g., monitoring and avoiding environmental seizure triggers), and managing their co-morbid conditions (e.g., anxiety, depression). The clinic-based Management Information Decision Support Epilepsy Tool (MINDSET) is a decision-support system founded on theory and empirical evidence. It is designed to increase awareness by adult patients (≥18 years) and their health-care provider regarding the patient’s epilepsy S-M behaviors, facilitate communication during the clinic visit to prioritize S-M goals and strategies commensurate with the patient’s needs, and increase the patient’s self-efficacy to achieve those goals.

**Methods:**

The purpose of this paper is to describe the application of intervention mapping (IM) to develop, implement, and formatively evaluate the clinic-based MINDSET prototype and in developing implementation and evaluation plans. Deliverables comprised a logic model of the problem (IM Step 1); matrices of program objectives (IM Step 2); a program planning document comprising scope, sequence, theory-based methods, and practical strategies (IM Step 3); a functional MINDSET program prototype (IM Step 4); plans for implementation (IM Step 5); and evaluation (IM Step 6). IM provided a logical and systematic approach to developing and evaluating clinic-based decision support toward epilepsy S-M.

## Introduction: Background and Rationale

Epilepsy is a neurological disorder involving recurrent seizures (1). It affects approximately 5 million people in the US (1). Epilepsy onset is not age dependent but incidence rates peak before 5 and after 60 years of age (2). Greater psychosocial impact is likely when seizure onset is in adolescence compared to younger ages (2). Epilepsy can have adverse social, physical, and psychological consequences, compromising a person’s economic and social future. The direct costs of epilepsy care were estimated to range from $8,412 to $9,287 in 2013 and were markedly higher for sub-populations with uncontrolled or refractory epilepsy, or co-morbidity (3).

### Epilepsy Self-Management (S-M)

People with epilepsy (PWE) have varied disease severity. Regardless, PWE are encouraged to manage their treatment and lifestyle to provide optimal quality of life. The Managing Epilepsy Well (MEW) Network defines epilepsy S-M as the “processes a person uses to optimize seizure control, to minimize the effects of having a seizure disorder, and to maximize quality of life in partnership with their health-care provider” (4, 5). This includes both S-M behaviors that are specific to epilepsy as well as S-M behaviors for chronic care that are applicable to most chronic conditions (2). Epilepsy specific S-M encompasses managing treatment (e.g., adhering to anti-seizure medication and clinical visit schedules), managing seizures (e.g., planning for, and responding to, seizure episodes), managing safety (e.g., monitoring and avoiding environmental seizure triggers), and managing co-morbid conditions (e.g., anxiety, depression). Chronic care S-M encompasses management of lifestyle issues (e.g., adjusting typical behaviors to avoid seizures and/or to mitigate their adverse consequences), partnering actively with the health-care team (e.g., information sharing), and pursuing independence (e.g., invoking support, resources, and services when needed) (2, 6). Knowledge and self-efficacy to perform S-M behavior are associated with epilepsy S-M (5, 7–10). S-M practice can be compromised by co-morbidities including depression, anxiety, and cognitive dysfunction. These can also act directly as internal precipitants of seizures (2, 11, 12). The emergence of S-M research in epilepsy has co-occurred with the development of the MEW Research Network. The development described in this paper occurred as a MEW Network collaborative project, supporting the Network’s long-term objective to increase the number of adequately tested epilepsy S-M programs available to health-care providers (HCPs) and members of the epilepsy community. The aim of the Network is to contribute to applied research targeting the priority recommendations from the CDC Epilepsy Program and Living Well with Epilepsy 2003 to promoting S-M (5, 13). The Network’s objectives are to: (1) “develop and implement a coordinated, applied-research agenda”; (2) conduct rigorous research that promote S-M and quality of life suitable for application in diverse settings including homes, communities, and clinics; and (3) to identify and collaborate with stakeholders outside of the network to implement these activities (5). The importance of S-M for PWE and programs available to assist S-M are discussed in the needs assessment section below.

### Patient and Provider Collaboration

The Institute of Medicine (IOM) report, Epilepsy Across the Spectrum, promotes patient-centered care for epilepsy and related co-morbidities, including collaborative approaches (2). Partnership between the HCP, including clinicians, nurse educators, and community health workers and the patient (including the patient’s family or significant others), is important in facilitating S-M adherence. Consistent with the patient-centered model of caring for people with a chronic disease HCPs are well positioned to help their patients in meet S-M goals (2).

Shared decision involves HCPs and patients making decisions together based on the best evidence available. This promotes a two-way communication that incorporates clinician expertise (i.e., disease, options, probabilities, and prognosis) and patient expertise (i.e., “preferences, values, attitudes to risk, and social circumstances”) (14). Dual participation enables the best solution when varied options are available. Prompting patients before the clinical encounter can result in better shared decision-making and enable the transfer of more salient information from the HCP (14). Patient care plans or action plans can be useful, allowing patients to consider individual preferences on options and treatment goals prior to discussion. Systematic review indicates that shared decision-making can lead to better patient treatment adherence (15).

Health-care providers need to be able to clearly communicate the risks associated with epilepsy, the importance of S-M, potential side effects to treatment options, and resources and services that are available (2). Patients need to determine if the type and frequency of their S-M behavior adherence is appropriate; decide on S-M goals that they perceive as important and doable; and determine how to accomplish these behaviors in everyday life. Adoption of S-M behaviors can be undermined due to poor patient-HCP communication and/or a discrepancy in the perceptions about the patient’s attitudes to, and S-M abilities, regarding their epilepsy. Conversely, by reinforcing patient S-M, HCPs can instill greater commitment to monitoring and improving behaviors (16, 17). There are challenges to effectively incorporating S-M assistance within a brief clinical visit that limits the time to assess a patient’s S-M needs and adequately address them (18, 19).

### Mobile Health (mHealth) Decision Support As an Intervention Channel

The IOM report also cited the need for new tools to enhance S-M decision-making (2). A decision-support system (DSS), broadly defined, is a tool to support the decision-making process. Typically, such tools are used in the context of less well-structured problems, enable the incorporation of varied models and analytic techniques, provide easy use by non-experts, and are flexible in accommodating changes in circumstances. Text- and video-based materials exist to assist patients and their HCPs in complex decision-making toward outcomes reflective of patient values and preferences (2, 13, 20–24). Electronic health (eHealth) applications are emerging that support daily S-M monitoring and decision-making for epilepsy (25). mHealth is a subset of eHealth that pertains to the “practice of medicine and public health supported by mobile devices such as mobile phones, tablet computers, and PDAs” (26). Clinic-based DSSs have focused on the technical aspects of diagnostic and pharmacologic decisions (27–33) and less on the personal or social aspects of patient care (34). Facilitating patient and HCP epilepsy S-M decision-making, therefore, represents a novel application of decision support.

The clinic-based Management Information Decision Support Epilepsy Tool (MINDSET) was developed to (1) engage adult patients with epilepsy (≥18 years) and their HCPs in managing therapy and lifestyle to prevent seizures and maximize quality of life (2), (2) provide easily followed goal-based action plans for patient decision support between clinic visits (35), and (3) document patient-centric quality indicators for epilepsy care (36, 37).

Management Information Decision Support Epilepsy Tool is a DSS founded on theory and empirical evidence. It is designed to increase awareness by adult patients and their HCP regarding the patient’s epilepsy S-M behaviors, facilitate communication during the clinic visit to prioritize S-M goals and strategies commensurate with the patient’s needs, and to increase the patient’s self-efficacy to achieve those goals.

### Interventional Mapping (IM)

Intervention mapping is a stepped framework to guide the development of behavioral change interventions that enable developers to systematically apply social and behavioral science theories (38). The 6 steps of IM are to (1) assess needs and develop a logic model of the problem, (2) develop matrices of behavioral change objectives for the program, (3) identify theory-based methods and practical applications to be applied in the program, (4) produce program components and materials, (5) plan for program adoption, implementation, and sustainability, and (6) plan for evaluation (38). IM is widely used to develop behavioral change interventions worldwide. A recent systematic review has demonstrated significant increase in the uptake of disease prevention behaviors associated with IM-based interventions when compared to placebo control groups (39). IM has been successfully applied in the domain of chronic disease S-M (39). However, few applications of IM have been reported in the context of managing epilepsy and, to our knowledge, none in the context of support for patient and provider epilepsy S-M decision-making.

The purpose of this paper is to describe the application of IM to develop and formatively evaluate MINDSET to be a clinic-based tool for adult patient (≥18 years) and provider decision-making regarding the patient’s S-M. Plans for subsequent efficacy evaluation are briefly described.

## Methods: IM

### The Development Timeline

IM steps 1 through 4 are the focus of this paper. Completion of these steps approximated 2 years of development time. The first 6 months of Year 1 involved completion of the logic model of the problem (IM Step 1) and defining program outcomes and objectives and the logic model of change (IM step 2). The remaining 6 months of year 1 involved program planning, developing the MINDSET design document (IM Step 3). The first 6 months of Year 2 involved producing a program prototype and the remaining 6 months of year 2 involved formative evaluation, including alpha- and usability-testing (IM step 4). Plans for implementation and evaluation (IM Steps 5 and 6) were commenced during the period of MINDSET formative testing.

#### IM Step 1: Logic Model of the Problem

Step 1 comprised establishing a planning group; conducting a needs assessment informed by the PRECEDE planning model that outlines the factors associated with the problem; defining the context of the intervention in terms of population, setting, and community; and stating program goals.

##### Task 1.1 Establish and Work with a Planning Group

Management Information Decision Support Epilepsy Tool development took place in collaboration with three neurology clinics varying in patient population, payer-base, epilepsy cases, and provider experience: Kelsey–Seybold Neurology Clinic (KS clinic) and their associated Education and Research Program, the Smith Clinic at Harris Health, and the University of Texas Physicians-Neurology Clinic (UT clinic). The clinics enabled access to patients and neurologists for a Patient-Provider Advisory Group (PPAG; described below) that provided ongoing input on MINDSET development through each step of the IM process and also provided a test-bed for formative assessment of MINDSET. These clinics were the test sites for the planned efficacy trial of MINDSET.

###### Collaborating Clinic Sites

The KS clinic operates within a large urban multispecialty medical organization comprising 21 clinics and over 325,000 diverse patients comprising primarily white (55%), African-American (23%), and Hispanic (19%) ethnicities who are mainly middle-class, employed, and with private insurance coverage primarily through HMO- or PPO-type plans. Patients with epilepsy are referred to the centralized KS neurology clinic. HCPs include general neurologists (*n* = 5), an epileptologist, and a nurse epilepsy specialist. The neurology department has an annual epilepsy case load of approximately 400 patients. The Epilepsy Education and Research Program was established at KS Clinic in 1987 with the goal to demystify epilepsy through patient and family education and training about epilepsy, its treatment, and management, and to develop and conduct research to improve the clinical management of epilepsy through participation in multicenter clinical drug trials and academic collaborations (3, 40, 41).

The *Smith Clinic at Harris Health* provides care to patients who are primarily Hispanic (40%), low-income, uninsured, and covered by Medicaid, and are referred from community health centers (*n* = 12) operated by a large public hospital system. Medical residents and students rotating through the clinic see up to 40 patients per clinic day under the supervision of attending faculty.

The UT clinic is a large urban multispecialty neurology clinic. Patients with epilepsy comprise white (56%), black (14%), Hispanic (4%), Asian (0.2%), and other/unknown (27%). Economic status and financial coverage for health care is diverse, predominantly commercially managed care (58%), and Medicare (31%). The clinic is a tertiary care referral center for neurological disorders, including the diagnosis of epilepsy and the management of difficult epilepsy.

###### Patient Provider Advisory Group

A PPAG was formed with representation from patients and HCPs from the three clinics and incorporated into the MINDSET research and planning team. The PPAG was consulted to review content (e.g., constructs, scales, and threshold scores for identifying “at-risk” patients); assess functionality, flow, and “look and feel;” test usability; and review evaluation plans. Patients for the advisory group were invited to join the PPAG by co-investigator clinicians and nurses on the basis of their being representative of the patient population, over 18 years of age, English speaking, engaged in epilepsy management issues, and interested in contributing to the field. The PPAG included three neurologists, one nurse educator, and eight patients with epilepsy. The PPAG met in a conference room at the KS Clinic. Patient members received an incentive payment of $30 per meeting.

##### Task 1.2 Conduct a Needs Assessment to Create a Logic Model of the Problem

Information gathered to inform the development of MINDSET was obtained through literature review, quantitative enquiry with the PPAG, empirical investigation of the association of S-M antecedents with PWE in Houston clinics, and clinic-based system task analysis (described in Step 4). The needs assessment was designed to inform a logic model of the problem, to provide background information on challenges experienced by PWE in epilepsy S-M, and the potential for technology to assist patient and HCP decision-making regarding epilepsy S-M.

###### Literature Review

A decision support tool for identifying patient S-M needs based on clinical, behavioral, and psychosocial variables requires identifying S-M behaviors and the clinical, behavioral, and psychosocial antecedents related to poor S-M as well as to identifying what other instruments/tools might be available in the field. To develop a logic model of the problem, the literature review addressed the medical management of epilepsy, epilepsy S-M behaviors, determinants of S-M behavior, and environmental factors associated with S-M. Data on the S-M interventions, DSSs in support of epilepsy management, and perceptions of PWE toward technology-based applications were also reviewed to understand the empirical and clinical context. Theories and models applicable to chronic disease management amenable to, or applied to, epilepsy were also reviewed, as were practice guidelines for epilepsy management. The research team developed problem statements, identified relevant electronic publication databases of Medline, PubMed, and PsychINFO, formulated database search strategies, and recommended an approach to synthesizing the literature. Data abstraction forms were developed and pilot-tested before they were used to abstract data from the identified relevant studies. Abstracted data were used to create evidence and information tables for expert review. We considered articles published in peer-reviewed journals, including review articles and surveys as well as practice guidelines. Abstracts, poster presentations, and editorial publications were excluded.

###### Medical Management and the Pathophysiology of Epilepsy

As with many chronic diseases, patients with epilepsy may undergo benign or malignant courses, but all will be affected significantly in some way (21, 42). Most patients with epilepsy undergo basic serological tests, EEG, and imaging studies, and have treatment initiated with a single anti-seizure medication (referred to, henceforth, by the common term anti-epilepsy drugs or AEDs) appropriate for the type of seizure, and age and gender of the patient. If the first agent does not control the seizures or has unacceptable toxicity, switching to a second or third appropriate agent occasionally provides better results. Some patients have seizures incompletely controlled with a single agent, but the addition of a second medication only allows a further 15% seizure control. The choice of a specific AEDs for a given patient is a fairly complex process, which needs to consider the individual’s tolerance for medication in general, seizure type, etiology of seizures, co-morbid conditions, concurrent medications, as well as non-medical factors such as employment and medication costs. Despite optimal pharmaceutical treatment, approximately 30% of patients will have recurrent seizures, and as many as 50% of patients with partial seizures will not attain complete seizure control with medication regimens. Patients who do not respond adequately to AEDs may be candidates for surgical treatment or other alternative regimens, including the ketogenic diet, vagal nerve stimulator, and control of precipitating factors (43). The pathophysiology of epilepsy varies between individual patients who may experience a number of different seizure types (e.g., generalized tonic-clinic seizures characterized by convulsions; absence seizures characterized by abrupt beginning and end, blank stares, and only a few second in duration; and complex partial seizures that are characterized by altered consciousness where there is no memory of the misplaced behavior demonstrated during the seizure) and varied stimulus onsets (43).

###### Epilepsy S-M Behaviors

**Figure 1:**
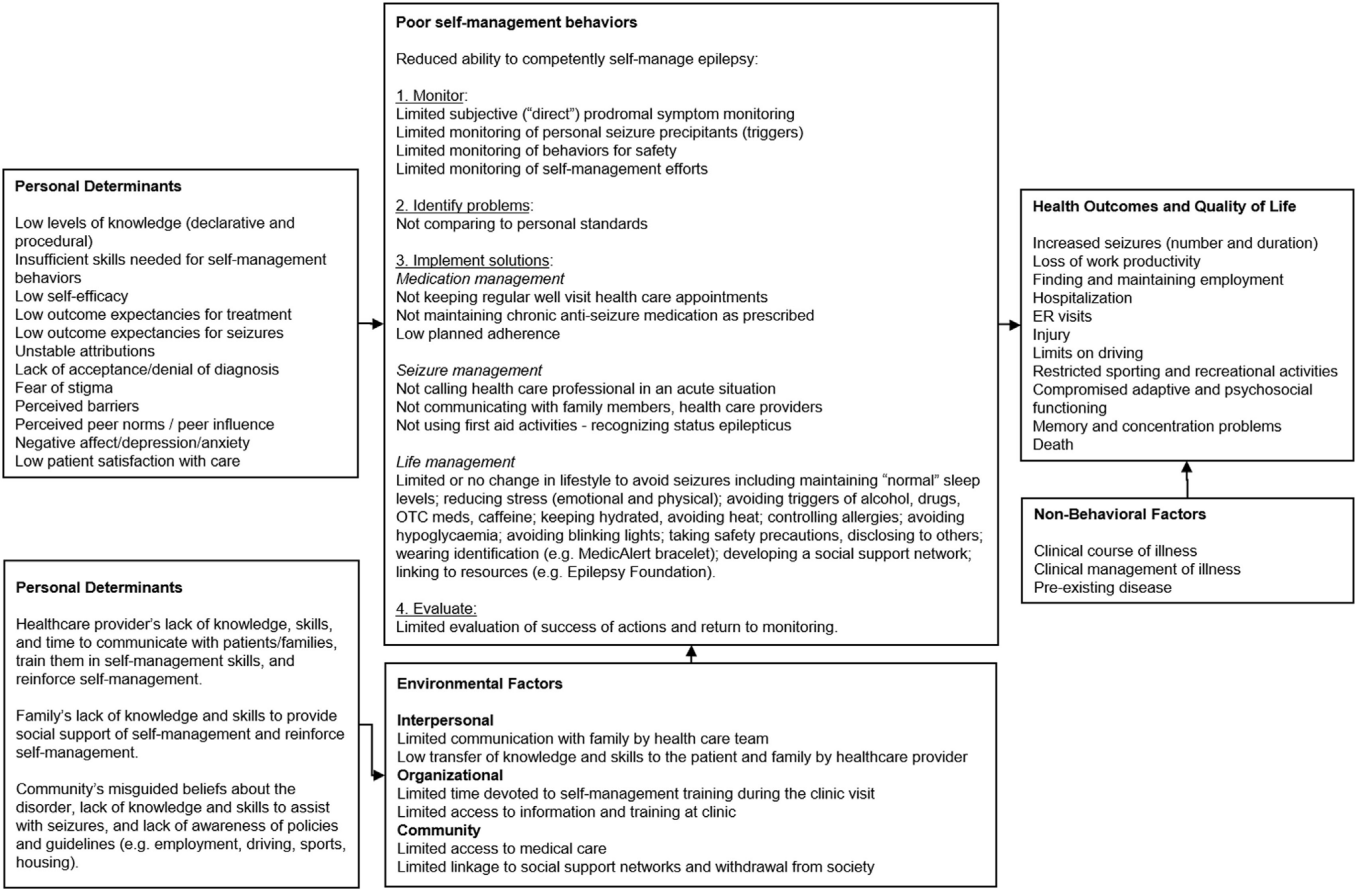
PRECEDE logic model of the problem for Management information Decision Support Epielpsy Tool (MINDSET) (101).

For PWE, S-M comprises a number of adaptive behaviors that may assist in lowering seizures (44). S-M for PWE refers to a number of adaptive behaviors that may assist in lowering seizures. In recent years patient S-M has received more attention (2, 20). Behavioral risk factors contributing to seizures and co-morbidities include lack of adherence to AEDs, failure to monitor and protect against seizure triggers, lack of safety management to minimize the adverse consequences of seizures, failure to adhere to clinical visit regimens, and failure to adjust lifestyle behaviors to minimize risk of injury (2, 6, 45) (Figure 1). Epilepsy co-morbidities are associated with poor S-M and include cognitive dysfunction, depression, suicidal ideation, death resulting from a seizure or *status epilepticus*, and sudden unexpected death in epilepsy (SUDEP). Medication management (adherence), safety behaviors (e.g., cessation of driving), and daily activities (e.g., maintaining sleep and reducing stress and exposure to triggers of seizure triggers) may lower seizure frequency. The focus of seizure control is management of AEDs. AEDs require strict adherence and, even with this, may not completely control seizure activity in 30% of epilepsy patients (46). Compounding this is low AED adherence. Review data for claims indicates 39% of patients do not take their prescribed regimen (47). Failure to adhere to prescription is associated with increased likelihood of hospitalizations and ER visits (47). Poor adherence is related to significant adverse health effects and increased mortality (48, 49). Uncontrolled seizures place “challenging demands” on PWE and their family and strongly predict low quality of life (50), being related to injury, limits on driving, and restrictions on sporting and recreational activities. Other S-M activities, apart from AED adherence, can increase mood and quality of life. For example, self-monitoring can increase awareness of prodromal (early) features of seizures (e.g., “mood and premonitory triggers of blurred vision, hunger, thirst, tiredness”) (44). Such self-prediction is associated with favorable mood and increased confidence in one’s ability to accurately predict seizures (51).

###### Personal Determinants of S-M Behavior

A range of factors provide antecedents for S-M behavior (Figure 1). Poor S-M could be due to the patient’s low levels of knowledge (declarative and procedural) and skill regarding epilepsy S-M behavior and goal setting, low self-efficacy or confidence to perform S-M behaviors, low outcome expectations (both in terms of causality of seizure onset as well as causality of treatment), and lack of attribution of S-M success to self-effort (particularly as this relates to patient control); lack of acceptance or denial of the diagnosis of epilepsy; fear of stigma related to epilepsy; perceived barriers to managing epilepsy, as well as unrealistic perceptions of how other PWE self-manage (2, 7–9, 11, 16, 52–62). Epilepsy is often associated with cognitive dysfunction, behavior problems, depression, and anxiety (12). Furthermore, seizures in epilepsy may be precipitated by psychological triggers such as stress and emotions such as anxiety and anger (12). Patient’s perceptions of, and satisfaction with, health services and clinical care are associated with health care utilization, an important aspect of S-M (2). Many PWE lack the behavioral capability to monitor and self-regulate behaviors that affect seizure susceptibility indicating a need for effective S-M programs (2). Such behaviors include AED adherence, exposure to environmental stimuli, overuse of drugs and alcohol, stress reduction, and ensuring adequate sleep (2).

###### Personal Determinants of the Environmental Factors

Interpersonal, organizational, and community factors impact PWE. Personal determinants of environmental factors involve HCPs, families, and the community (Figure 1) (2). Epilepsy management is compromised when families lack knowledge and skills for providing support for S-M, and HCPs lack the skills to effectively communicate with patients and families to train them on, and reinforce them for epilepsy management behaviors (2, 12). This is compounded by the general community’s misguided beliefs about epilepsy, lack of knowledge and skills to assist with seizures and support management, and lack of awareness of policies and guidelines regarding supporting PWE in important life functions, including employment, driving, sports, and housing (2).

###### S-M Interventions

Until recently, there were few evidence-based epilepsy educational programs (63). Reported results were encouraging. In a study among 100 adults with epilepsy with a two-day psycho-educational program (Sepulveda Epilepsy Education) significant effects were demonstrated that included greater serum AED levels (indicating better drug adherence), decreased use of hazardous medical S-M practices, greater understanding of epilepsy, and decreased fear of seizures in treatment group compared to the comparison group (64). A study among adult Nigerian patients, a two-day modular didactic psycho-educational program focused on adjusting to epilepsy and related psychoneurotic traits, depression, and stigma demonstrated significant improvement in knowledge of epilepsy, neurotic disorders, and depression, in the treatment group compared to the comparison group (65). A modular didactic educational program (MOSES, Modular Service Package Epilepsy) evaluated on a sample of 242 participants, demonstrated greater tolerance of AEDs, fewer side effects, improved knowledge and coping, and greater satisfaction with therapy in the treatment group compared to the comparison group (56). Interventions were mainly psycho-educational with minimal focus on S-M as previously defined. Recent evidence-based interventions that target S-M behaviors and/or co-morbidities for adults (≥18 years) include WebEase (Web Epilepsy Awareness, Support, and Education), UPLIFT (Using Practice and Learning to Increase Favorable Thoughts), PEARLS (Program to Encourage Active Rewarding Lives), HOBSCOTCH (HOme Based S-M and COgnitive Training CHanges lives), and PACES in Epilepsy (Program of Active Consumer Engagement in S-M) (66). WebEase is a self-paced online website where PWE can choose from among medication, stress, sleep, and personal tracking diary modules that provide tailored activities for learning, self-assessment, and goal setting (assessed at 1-week intervals). A national RCT (*n* = 148) demonstrated significant improvement in self-efficacy and medication adherence for those using WebEase (67). Other interventions have greater focus on co-morbidities of depression [UPLIFT (68), PEARLS (69)] and subjective memory complaints (HOBSCOTCH) (70) or a niche priority population consumers with active epilepsy (with seizures occurring within the last year) (PACES) (71). Tools to optimize decision-making for S-M for patients and providers within the clinic visit had not been reported.

###### Decision Support and eHealth Applications in Epilepsy Management and Patient Perceptions

The Centers for Disease Control and Prevention (CDC) Epilepsy Program supported the development of e-Tools as one of several approaches to address the gap in available epilepsy S-M tools (1). This vehicle has the potential to overcome barriers to care that PWE face such as lack of transportation and stigma (1). Despite this, a review of the literature on informatics applications for epilepsy management revealed an only recently emerging research effort (25). Of the 68 studies reviewed in the domains of patient monitoring and prevention, education, and therapy or guideline application most were descriptive (describing models, system development, or system installation) with only eight studies testing effectiveness (the impact on patient or provider behavior) using prospective design (25). PWE are well positioned to use emerging eHealth applications in epilepsy S-M. Over 50% of PWE have access to the Internet in a variety of settings (i.e., home, work, school, library) (72–74) and a recent cross-sectional study (19) with adult PWE (*n* = 183) indicated that most participants had access to computers and the Internet (95 and 60%, respectively) and used them to find health information (99 and 57%, respectively). Participants reported “searching for general information on epilepsy (43%), medication (30%), specific types of epilepsy (23%), and treatment (20%)” and most reported that they “likely would use an Internet-based S-M program to help control their epilepsy” (19). Counter-balancing this is needs assessment survey data of adults with epilepsy in the Pacific Northwest (*n* = 165) (75, 76) indicating that a majority of patients prefer in-person (individual or group) program delivery, reinforcing the importance of this over purely distance delivery (phone or Internet).

While the patients in the PPAG were veteran self-managers and mostly exhibiting good seizure control they expressed frustration regarding their relationship with their HCP. This applied particularly to patients from a large inner city community health clinic where brief “face time” with clinicians and frequent and consistent turnover of fellows hampered the development of an ongoing therapeutic relationship. They considered a tablet-based DSS within the context of a clinic visit as a positive addition. Many were using the internet to acquire information on epilepsy and most wanted greater communication with their HCP. There was general agreement for the potential of the epilepsy DSS as a clinical tool in facilitating patient-provider communication.

###### Review of Selected Theories, Models, and Practice Guidelines for Chronic Disease Management

Social cognitive theory (SCT) and self-regulation models (77–79) were consistently reported in the literature in the context of S-M of epilepsy and other chronic diseases (80, 81) and associated with key psychosocial determinants, including knowledge, outcome expectations, and self-efficacy and skills previously described. A tenet of SCT is that behavior is determined by the interaction of personal, environmental, and behavioral influences (77). Personal influencers include cognitions, such as personal values, beliefs, skills, outcome expectations, and self-efficacy. Environmental influencers include social or physical factors (e.g., influential role models, social or normative support). *Self-regulation* is a potent SCT concept for organizing health education in the management of chronic health disorders (82, 83). It comprises primary sub-functions of behavior self-monitoring (including antecedents and consequences); judgment of one’s behavior in comparison to optimal personal standards and environmental circumstances; and self-reaction (behavior to rectify drifts from optimal S-M) (84). The categorization of S-M behaviors in Figure 1 are informed by this self-regulation framework (Figure 1). The term self-regulation refers to both the patient’s management of his/her own care and the transfer of S-M tasks to the patient by the HCP as appropriate. Self-regulation has the potential of improving the patient’s autonomy and increasing adherence to medical regimens, which can improve medical outcome. Self-regulation necessitates a more prominent role of the patient in first determining, and then monitoring, behaviors and environment, and then modifying therapeutic regimens accordingly in collaboration with the HCP. Self-efficacy and outcome expectations have been described as determinants of epilepsy S-M behavior (10, 16).

The 5-A’s model of behavior change (81), quality-of-care criteria, and clinical guidelines for epilepsy (13, 31, 32, 85); informed MINDSET’s scope, components, and relevance within a clinical context (described in Step 3). Motivational enhancement therapy protocols (86) provided a means of eliciting decision-making within an mHealth program. Both motivational interviewing and shared decision-making supports the ethical principle of self-determination (87) Motivational enhancement protocols used to elicit movement toward behavioral change had been used in previous decision-support studies (88, 89).

###### Empirical Study of S-M Determinants in the Target Population

To collect additional data on determinants of poor epilepsy S-M in their priority population, the planning team conducted surveys with PWE receiving care at two clinics in the Houston area (*n* = 238) (10). The objective was to examine variation in S-M across diverse patient populations and explore the association between personal psychosocial factors (knowledge, self-efficacy, depression, and stigma) with S-M. A cohort of 437 patients previously enrolled in the CDC-funded Epilepsy Care and Outcomes Study (41) completed a 45-minute S-M survey within the context of their regular clinic visit. The survey comprised scales previously reported in epilepsy-related research, including the Epilepsy S-M Scale, Epilepsy Knowledge Scale, Epilepsy Self-efficacy Scale, Outcome expectations, Shared Control portion of the Multidimensional Desire for Control Scale, Personal Resource Questionnaire 85 Part 2, Center for Epidemiologic Studies Depression Scale, and Modified Parent Stigma Scale, and Patient Satisfaction Questionnaire-III. The justification for these scales was recent research that had focused on assessing the association of these factors and epilepsy-related S-M behaviors. DiIorio et al. (90) determined the association of the assistance aspect of social support with regimen-specific support (41). Self-efficacy was significantly associated with outcome expectancy and anxiety in the predicted directions and anxiety was significantly negatively associated with S-M (90). DiIorio et al. determined that self-efficacy and patient satisfaction explained the most variance in medication management (16). Self-efficacy was associated with social support, stigma, outcome expectations, and depressive symptoms. Stigma was associated with depressive symptoms (16). The overall fit of the model was improved by adding the direct association between stigma and outcome expectations for seizures to S-M (90). DiIorio et al. (91) identified depressive symptoms and seizure severity as significant antecedents of self-efficacy for epilepsy S-M. Also significant were predictors of social support and stigma (91). Self-efficacy, social support, depression, and perceived stigma were significantly related to S-M regardless of demographics, seizure frequency, or socio-economic status (p < 0.05). These findings suggested that the difficulties with S-M faced by many patients with epilepsy are similar irrespective of a patient’s background or characteristics and that the types of strategies to improve S-M appear similar regardless of population heterogeneity.

##### Task 1.3 Describe the Context for the Intervention, Including the Population, Setting, and Community

Management Information Decision Support Epilepsy Tool development was modestly focused on application for patients in the collaborating clinics (previously described). The heterogeneity offered in the clinic type (HMO, community clinic, and teaching hospital) and the patient population (demographics and epilepsy type) provided an excellent test-bed for development.

The family, significant others, and community sentiment regarding epilepsy were important environmental influences (Figure 1). Given the clinical setting, the priority environmental focus was the HCP. A caregiver component was considered, and though valid, represented an extension of project scope without universal relevance to PWE who may lack this social network, who are not accompanied to clinic visits, and who are not ready to involve others in their S-M. Broader community influencers, while important, were also outside the scope of the project.

##### Task 1.4 State Program Goals

Goals for MINDSET were to influence patient S-M behavior and to influence the mediating patient-provider communication regarding S-M. Respective patient and provider goals for MINDSET included:

1. Patients with epilepsy who use the MINDSET S-M DSS in the context of their usual clinic visit for three consecutive clinic visits over a 9-month period will report at least three fewer “at-risk” S-M behaviors (assessed by the Epilepsy S-M scale) compared to patients who do not use MINDSET.
2. HCPs who use the MINDSET S-M DSS in the context of their usual clinic visit will focus discussion on at least 3 “at-risk” S-M behaviors (assessed by the Epilepsy S-M scale) at every visit with every patient using MINDSET.

#### IM Step 2: Program Outcomes and Objectives—Logic Model of Change

Step 2 comprised: identification of expected outcomes, performance objectives (POs), and determinants for the behavior and environment; the development of matrices of change objectives; and the construction of a logic model of change for the program. This step enabled the triangulation of data obtained in Step 1 (from theory, empirical findings, and participant involvement) to inform a logic model of change.

##### Task 2.1 State Expected Outcomes for Behavior and Environment

###### Expected Behavioral Outcomes

Management Information Decision Support Epilepsy Tool was designed to positively impact S-M behavior for epilepsy that encompassed three domains: Medication management, seizure management, and lifestyle management.

The expected behavioral outcomes for PWE related to each domain were as follows:

- Take AEDs as prescribed by the physician (medication management).
- Prepare for, and respond to, seizure episodes (seizure management).
- Alter behaviors to avoid seizure onset and seizure-related injury (lifestyle management).

Targeted health and quality of life outcomes included decreased seizures (number and duration) and AED side effects and improved daily functioning resulting in improved work productivity, less injury, and reduced ER visits, hospitalization, or death attributable to epilepsy (Figure 1).

###### Expected Environmental Outcomes

Management Information Decision Support Epilepsy Tool was designed for use in the clinic visit so S-M assessment and intervention needed to become a minimally invasive component of the clinic flow. Rather than manipulate varied clinic environments (which would be prohibitive when considering future dissemination), the environmental outcome focused on the interpersonal level of the HCP (neurologist and nurse educator). Therefore, the environmental outcome was focused at a personal level:

- HCP and/or nurse educator will support PWE to self-manage their condition.

##### Task 2.2 Specify POs for Health-Promoting Behavior and Environmental Outcomes

###### POs for Epilepsy S-M

**Figure 2:**
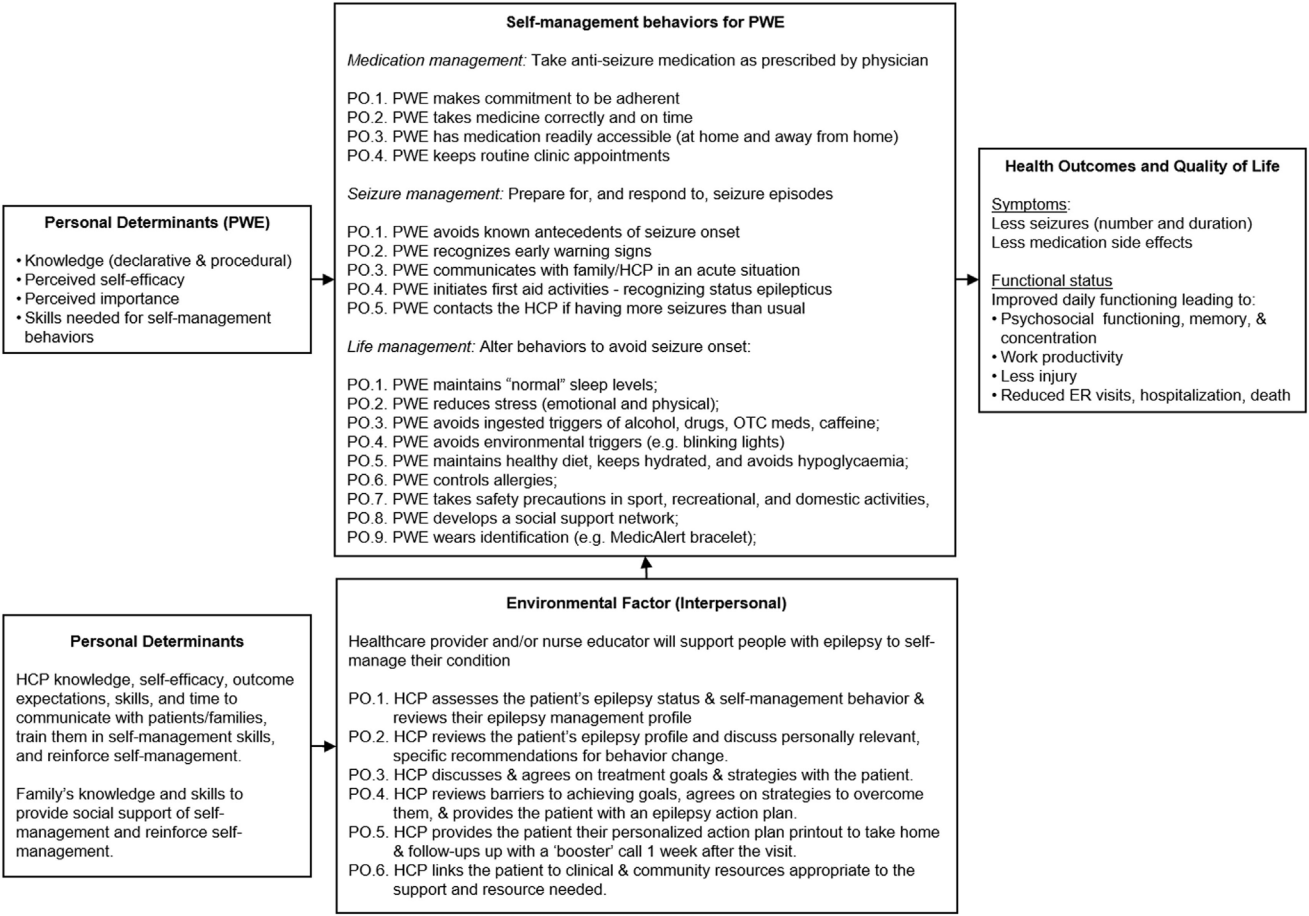
PRECEDE logic model of change for MINDSET (101).

Performance objectives were described for each S-M outcome: medication management, seizure management, and lifestyle management. These are listed in Figure 2 and were drawn mainly from review of existing literature and S-M measurement instrument domains (7). MINDSET and the patient action plan alert both the patient and HCP when change in S-M behavior is needed and cues them to decide on S-M priorities or goals based on available evidence and to agree on how best to achieve these S-M changes. The action plan provides the patient with an ongoing resource outside of the clinic visit on priority S-M performance objectives and strategies to achieve them (described further in Task 4.3 below).

###### POs for HCPs

The environmental focus for MINDSET was for the HCP to support S-M awareness raising and skills training (Figure 2). This included identification and review of S-M problems, and discussion with the patient to develop agreed upon S-M behavioral goals. The HCP’s behaviors were structured in accordance with the 5 A’s model and included requesting the patient complete data input into MINDSET, reviewing their epilepsy management profile, and acknowledging status (ASSESS); reviewing the patient’s epilepsy profile, reinforcing past management successes, and discussing personally relevant, specific recommendations for behavior change (ADVISE); reaching agreement on treatment goals with the patient (AGREE); reviewing barriers to achieving goals and agreeing on strategies to overcome them, and providing the patient with an action plan (ASSIST); reviewing epilepsy S-M change at each visit by comparing MINDSET epilepsy profile with that of the previous visit, arranging referrals appropriate to existing co-morbidities in the patient profile, and linking patients to appropriate community resources to provide the support needed (ARRANGE). The briefness of clinic encounters made it challenging to adequately review S-M and such assessments had lacked formality. There was opportunity for relative improvement in the HCP *modus operandi* as it pertained to S-M intervention.

##### Task 2.3 Select Determinants for Behavioral and Environmental Outcomes

Information obtained from the needs assessment phase (Step 1) and Step 2 literature review informed the specification of determinants for the POs. After reviewing findings from the empirical literature, SCT and self-regulation models, motivational enhancement therapy, and our own formative research (Task 1.2 above), we identified knowledge, self-efficacy, perceived importance, and skills as important and changeable determinants of epilepsy S-M for PWE (Table 1). Similarly, we identified knowledge, self-efficacy and skills, and outcome expectations as important and changeable determinants of the HCP’s behavior (Table 2).

**Table 1:** Example cells from the matrix of change objectives for patient behavior.

Behavioral Outcome: people with epilepsy (PWE) will take AED (ASM) as prescribed by physician			

Performance objectives (POs)	Determinants		
	Knowledge	Perceived importance	Perceived Self-efficacy and skills
PO.1. PWE makes commitment to be adherent	K1i. Describe how ASMs work K1ii. List consequences of non-adherence K1v. State reasons for taking meds as prescribed (will improve/maintain health, reduce likelihood of seizures, reduce likelihood of accidents or hospitalization)	PI1. State that it is important to take meds as prescribed to improve and maintain health status	SE/S1i. Express confidence and demonstrate ability to commit to ASM adherence SE/S1ii. Express confidence and demonstrate ability to understand how meds work

PO.2. PWE takes medicine correctly and on time	K2i. Describe why, how, and when to take meds correctly (name of pill, time, # pills, with/without food) K2ii. List situations that make taking meds on time difficult K2iii. List cues to action (memory aids) for taking meds correctly (e.g., by toothbrush, pill box, at mealtimes) K2iv. List ways to take meds discretely either at home or away from home K2v. Describe why and how to correctly make up for a missed dose(s) K2vi. State reasons to talk with physician if missing doses K2vii. List side effects	PI2 State that it is important to take meds correctly to improve and maintain health status	SE/S2i. Express confidence and demonstrate ability to take meds as prescribed SE/S2ii. Express confidence and demonstrate ability to take meds discretely if needed SE/S2iii. Express confidence and demonstrate ability to use cues/memory aids SE/S2iv. Express confidence and demonstrate ability to make up a missed dose(s) correctly SE/S2v. Express confidence and demonstrate ability to overcome side effects

PO.3. PWE has medication readily accessible (at home and away from home)	K3i. List personal medications K3ii. Lists places to store medication at home K3iii. List ways to carry medication when away from home K3iv. State how often prescription needs to be refilled K3v. If living alone, state how to refill prescription	PI3i. State that it is important to have medication readily available to reduce the likelihood of missing doses PI3ii. State that it is important to plan ahead to refill prescriptions to ensure constant supply of meds	SE/S3i. Express confidence and demonstrate ability to store medication appropriately at home SE/S3ii. Express confidence and demonstrate ability to carry medication outside of home SE/S3iii. If living alone, express confidence and demonstrate ability in filling prescription on time

PO.4. PWE keeps routine clinic appointments	K4i. State date/time of next appointment	PI4i. State that it is important to keep appointments so that the physician will be better able to monitor health and how well meds are working	SE/S4i. Express confidence and demonstrate ability in recording date/time of the next appt. and in keeping scheduled clinic appointments.

**Table 2:** Example cells from the interpersonal environment matrix for 0healthcare providers (HCPs).

Interpersonal outcome: HCP will support people with epilepsy (PWE) to self-manage their condition				

Performance objectives (POs)		Determinants		
		Knowledge	Outcome expectations	Self-efficacy and Skills
ASSESS PO.1. HCP assesses the patient’s epilepsy status and S-M behavior and reviews their epilepsy management profile	PO.1.i. Assess patient’s epilepsy status, including seizure history, medication history, side effects, compliance, and barriers	K1i. Describe how to assess the patient’s epilepsy status	OE1i. Expect that determining the patient’s epilepsy status leads to more salient treatment goals and better control of epilepsy	SE/S1i. Express confidence and demonstrate ability to interpret the patient’s status

	PO.1.ii. Assess patient’s S-M for seizure, medication, and lifestyle S-M	K1ii. Describe how to assess the patient’s epilepsy S-M behaviors	OE1ii. Expect that determining the patient’s epilepsy S-M leads to more salient S-M goals and better control of epilepsy	SE/S1ii. Express confidence and demonstrate ability to interpret the patient’s S-M

	PO.1.iii. Assess patient’s attitudes (importance and confidence) regarding S-M behaviors	K1iii. Describe how to interpret the patient’s perceived importance and self-efficacy to prioritize S-M goals	OE1iii. Expect that determining the patient’s perceived importance and self-efficacy for epilepsy S-M leads to more salient S-M goals and better control of epilepsy	SE/S1iii. Express confidence and demonstrate ability to interpret the patient’s perceived importance and self-efficacy

	PO.1.iv. Provide patient with personalized feedback on epilepsy status and S-M for review	K1iv. Describe how to ensure the patient has access to an action plan and how to print this for the patient	OE1iv. Expect that providing the tailored action plan to the patient for review will lead to more salient S-M goals and better control of epilepsy	SE/S1iv. Express confidence and demonstrate ability to be able to ensure the patient has access to an action plan and how to print this for the patient

ADVISE PO.2. HCP reviews the patient’s epilepsy profile and discusses personally relevant, specific recommendations for behavior change	PO.2.i. Relate patient symptoms or lab results to their behavior, recognizing patient’s culture or personal illness model	K2i Describe how including patient’s input in goal setting leads to greater adherence to the treatment plan	OE2i. Expect that creating patient treatment goals leads to better control of epilepsy	SE/S2i. Express confidence and demonstrate ability to determine appropriate treatment goals from patient information

	PO.2.ii. Inform patient that management is more than just taking medications	K2ii List reasons to treat epilepsy as a chronic illness	OE2ii. Expect that explaining S-M goals for epilepsy management will help the patient to achieve the outcomes described	SE/S2ii. Express confidence and demonstrates ability to be able to persuade patients that better function is possible when epilepsy is well treated

	PO.2.iii. Provide specific, documented behavior change advice (action plan) in the form of a prescription	K2iii Show familiarity with the action plan	OE2iii. Expect that using the action plan will enable patient to better manage epilepsy	SE/S2iii. Express confidence in being able to use plan at each visit

AGREE PO.3. HCP discusses and agrees on treatment goals and strategies with the patient	PO.3.i. Review with patient prioritized goals in the patient’s action plan	K3i. Describe how to review prioritized goals in the patient’s action plan	OE3i. Expect that reviewing prioritized goals in the patient’s action plan leads to greater adherence to the action plan	SE/S3i. Express confidence and demonstrate ability to review prioritized goals in the patient’s action plan

	PO.3.ii. Discuss and agree on specific goals to achieve by the next visit	K3ii. Describe how to include patient’s input in goal setting for shared decision-making	OE3ii. Expect that agreeing and meeting S-M goals will lead to better control of epilepsy	SE/S3ii. Express confidence and demonstrate ability to discuss and agree on appropriate treatment goals with the patient.

	PO.3.iii. Review recommended strategies with the patient needed to achieve targeted goals	K3iii. Describe how to include patient’s input in strategies for shared decision-making.	OE3iii. Expect that agreeing on strategies to meet S-M goals will lead to a greater chance of achieving those goals	SE/S3iii. Express confidence and demonstrate ability to review strategies to achieve S-M goals with the patient

	PO.3.iv. Review barriers to meeting S-M goals: Ask patient, “What are your most challenging barriers?,” recognizing physical, social and economic barriers	K3iv Describe how to review barriers to S-M goals using the action plan	OE3iv. Expect that reviewing barriers to S-M goals will lead to better S-M practice	SE/S3iv. Express confidence and demonstrate ability to be able to review barriers to S-M goals using the action plan

ASSIST PO.4. HCP reviews barriers to achieving goals, agrees on strategies to overcome them, and provides the patient with an epilepsy action plan	PO.4.i. Help patient develop strategies to address barriers to change (write on Action Plan form) (ask is there anything that would prevent you from doing these strategies?)	K4i. Describe how to review barriers and elicit patient’s input in strategies to overcome barriers	OE4i. Expect that listing barriers and strategies to overcome them will lead to a greater chance of achieving S-M goals	SE/S4i. Express confidence and demonstrate ability to determine barriers and list strategies to overcome them

	PO.4.ii. Refer patient to evidence-based education or behavioral counseling – individual or group	K4ii. Describe how to refer the patient to evidence-based education or behavioral counseling	OE4ii. Expect that referring the patient to evidence-based education or behavioral counseling will lead to a greater chance of achieving S-M goals	SE/S4ii. Express confidence and demonstrate ability to refer the patient to evidence-based education or behavioral counseling

	PO.4.iii. Elicit patient’s views and plans regarding potential resources and support within family and community	K4iii. Describe how to elicit the patient’s views and plans regarding family support	OE4iii. Expect that eliciting the patient’s views and plans regarding family support will lead to a greater chance of achieving S-M goals	SE/S4iii. Express confidence and demonstrate ability to elicit the patient’s views and plans regarding family support

ARRANGE	PO.5. HCP provides the patient with their personalized action plan printout to take home and follows-up with a “booster” call 1 week after the visit.	K5. Describe the process to provide the action plan and conduct a follow-up booster	OE5. Expect that providing the action plan and booster follow-up call will lead to better epilepsy S-M behavior	SE/S5. Express confidence and demonstrate ability to action plan and follow-up booster call

	PO.6. HCP links the patient to clinical and community resources appropriate to the support and resource needed	K6. Describe the process to link patients to clinical and community resources	OE6. Expect that linkage to clinical and community resources tailored to patient needs will lead to better epilepsy S-M behavior	SE/S6. Express confidence and ability to provide linkage to clinical and community resources

##### Task 2.4 Construct Matrices of Change Objectives

Matrices were developed that cross-referenced behavioral POs with determinants to produce change objectives. The resulting cells of each matrix contained change objectives that stated what needed to change about a specific determinant (e.g., self-efficacy) for the patient to achieve a specific PO. Change objectives were produced for each relevant cell of the matrix. Example cells from the matrix for adherence to the prescription plan for AEDs are provided in Table 1. Similarly, a matrix was developed to describe the behaviors to be engaged in by the HCP that incorporated the MINDSET action plan into the clinic encounter (Table 2).

##### Task 2.5 Create a Logic Model of Change

A logic model provided an understanding of the types of functional components MINDSET would need to provide to impact both the patient’s S-M behaviors as well as the HCP-patient discussion of S-M in the clinic visit (Figure 2).

#### IM Step 3: Program Plan

Step 3 comprised the generation of MINDSET’s scope and sequence, the choice of theory and evidence-based methods, and the design of practical applications to deliver change methods.

##### Task 3.1 Generate Program Themes, Components, Scope, and Sequence

The theoretical framework for MINDSET is based in SCT (77), self-regulation models (77, 78) the 5-A’s model of behavioral change (92), motivational enhancement therapy (86), quality-of-care criteria and clinical guidelines for epilepsy (13, 33, 34, 83), and formative studies (10, 93) drawn from the review of literature. The literature reviewed in Step 1 on decision support and S-M in epilepsy was particularly helpful in informing methods (10, 25, 94, 95).

The challenge was to develop a program to be able to fulfill five functional objectives involving both the patient and provider, without disrupting the flow of a typical clinic visit:

1. Increase patient awareness about their S-M behaviors.
2. Provide immediate feedback on S-M behaviors.
3. Provide a profile of the patient’s S-M behavior for the HCP.
4. Provide tailored S-M behavioral goals for the patient and HCP, including a printable S-M Action Plan.
5. Increase the potential for patient-provider communication of S-M problems and goal setting.

Management Information Decision Support Epilepsy Tool’s scope was contained to only relevant data necessary for the visit so as to not unduly intrude on the timing of events in the clinic flow and to not over-burden the patient. These objectives and our observation of the natural clinic flow suggested the scope and sequence of MINDSET. It was possible for the patient to enter and review their data in MINDSET in the waiting room prior to their visit, and then to provide this profile and the tailored action plan to the HCP for review and discussion in the clinic visit. MINDSET’s scope and sequence are more fully detailed in Step 4.2 below. The original working title for the program was “Brainstorm.” The PPAG advised against this title. While the notions of epilepsy as a brain-related disorder and thinking about management are apparent in this title the term “brainstorm” also has connotations with the erratic neural activity of a seizure and was considered too provocative by patients and providers. The MINDSET acronym, Management Information and Decision Support Epilepsy Tool, offered two contextually related meanings, that of the cognitive profile of the patient explored in the retrospective data input phase, and of “setting” one’s mind which relates to the prospective action plan phase.

##### Task 3.2 Choose Theory and Evidence-Based Change Methods

###### Individual Behaviors

Theoretical and empirically based methods for S-M education, included chunking of information into a meaningful framework of S-M domains, self-assessment of S-M behaviors, feedback of a S-M profile to the patient to give an assessment of their S-M status, reinforcement for behavioral successes, goal setting to address those behaviors that were a problem for S-M, tailoring of goals based on the patient’s individual profile, advance organizers and cue altering for S-M using behavioral strategies, self-monitoring of behaviors and environment, and facilitation and linkage to care/support as needed (Table 3). The research team selected methods based on (1) our previous work in decision support of chronic disease (96) and technology-based behavioral change interventions founded in self-regulation frameworks within varied health domains (97–99), (2) empirical evidence for use to impact the target determinants (exemplified in Table 3), and (3) the pragmatics of use in a tablet-based program. These methods and their related practical applications (Task 3.3) could all be delivered through repeated exposure to the MINDSET intervention in clinic visits over time. Their operationalization within MINDSET is described in Task 4.1 below.

**Table 3:** Example of methods and practical applications used in MINDSET to impact the determinants (knowledge, self-efficacy, perceived importance, and skills) for adhering to prescribed medications.

Behavioral outcome: people with epilepsy (PWE) will take anti-seizure medicine as prescribed by physician.Performance objective (PO) 2: PWE will take medications correctly and on time			

#	Objective	Method	Practical applications
	**Knowledge**		
1	K2iii list cues to action for taking meds	Chunking	Epilepsy management is categorized into 3 domains to enable the patient to organize what is a complex array of behaviors. The domains are management for seizures, medication, and lifestyle. Cues to action for taking meds, therefore, occurs within the domain if medication management
3		Feedback, Information transfer, Consciousness raising	The patient profile and action plan indicate the patient’s adherence status including “at-risk” medication management behavior, and how this has changed since the last visit (improved, worsened, no change), barriers to medication taking, self-efficacy, and importance
4		Reinforcement and remediation	The profile and action plan provide remediation that stresses the importance of medication management behaviors (e.g., strategies)
6		Tailoring	The patient profile and action plan are tailored to provide a list of S-M goals salient to the patient (based on data input) based on assessment of importance and self-efficacy. If the patient rates the medication adherence behavior as important and s/he has low efficacy to perform this behavior then the adherence behavior will be listed as a higher priority in the action plan
8		Advance organizing	The patient profile provides advice boxes and the action plan provides bulleted strategies on how to list cues to action for medication adherence
9		Cue to action	A cue is provided for the patient to discuss the medication adherence goal with the HCP during the clinic visit and a list of strategies related to memory aids is provided
	**Self-efficacy and skills**		
10	SE2iii express confidence in ability to use cues/memory aids	Reinforcement and remediation	The profile and action plan provide reinforcement messages (congratulatory statements) to patients who have no flagged medication management behaviors
			The profile and action plan provide remediation stressing the importance of medication management behaviors
11		Goal setting	If medication adherence behavior is flagged as “at-risk” then this behavior is reframed in the action plan as a S-M goal
12		Tailoring	The patient profile and action plan are tailored to provide a list of S-M goals salient to the patient (based on data input) based on assessment of importance and self-efficacy. If the patient rates the medication adherence behavior as important and s/he has low efficacy to perform this behavior then the adherence behavior will be listed as a higher priority in the action plan

13	S2iii demonstrate ability to use memory aids	Planning coping responses	Patient and HCP review and discuss causes (barriers) for medication non-adherence and review the patient profile and action plan for recommended strategies
			Patient and provider agree on the patient’s commitment to the medication adherence goal
14		Cue altering	Patient and HCP rehearse specific strategies and patient initiates cues to ensure adherence. For example, keeping a pill box in toiletry bag to cue packing meds before a trip and tagging refill dates on work schedules
15		Self-monitoring	Patient maintains a record of medication adherence
16		Facilitation/Linkage to care/support	Patient is linked to resources (e.g., Epilepsy Foundation) for more strategies
17		Repeated exposure	MINDSET is provided at each clinic visit
	**Importance**		
18	PI2 state that it is important to take medications correctly to improve and maintain health status	Self-assessment	The patient inputs information on his/her medication adherence and medication management behavior and, if adherence is a problem, barriers to medication taking, self-efficacy, and importance
19			
20		Reinforcement and remediation	The profile and action plan provide reinforcement messages (congratulatory statements) to patients who have no flagged medication management behaviors through
			The profile and action plan provide remediation stressing the importance of medication management behaviors
21		Goal setting	If medication adherence behavior is flagged as “at-risk” then this behavior is reframed in the action plan as a S-M goal.
22		Tailoring	The patient profile and action plan are tailored to provide a list of S-M goals salient to the patient (based on data input) based on assessment of importance and self-efficacy. If the patient rates the medication adherence behavior as important and s/he has low efficacy to perform this behavior then the adherence behavior will be listed as a higher priority in the action plan

###### Clinic Environment

**Figure 3:**
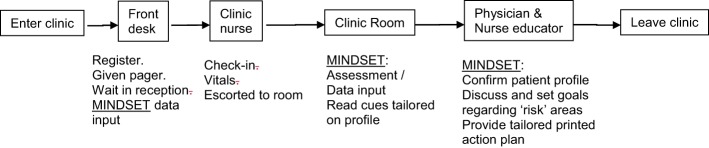
Management Information Decision Support Epilepsy Tool (MINDSET) use within the clinic visit and top-level flow (101).

**Figure 4:**
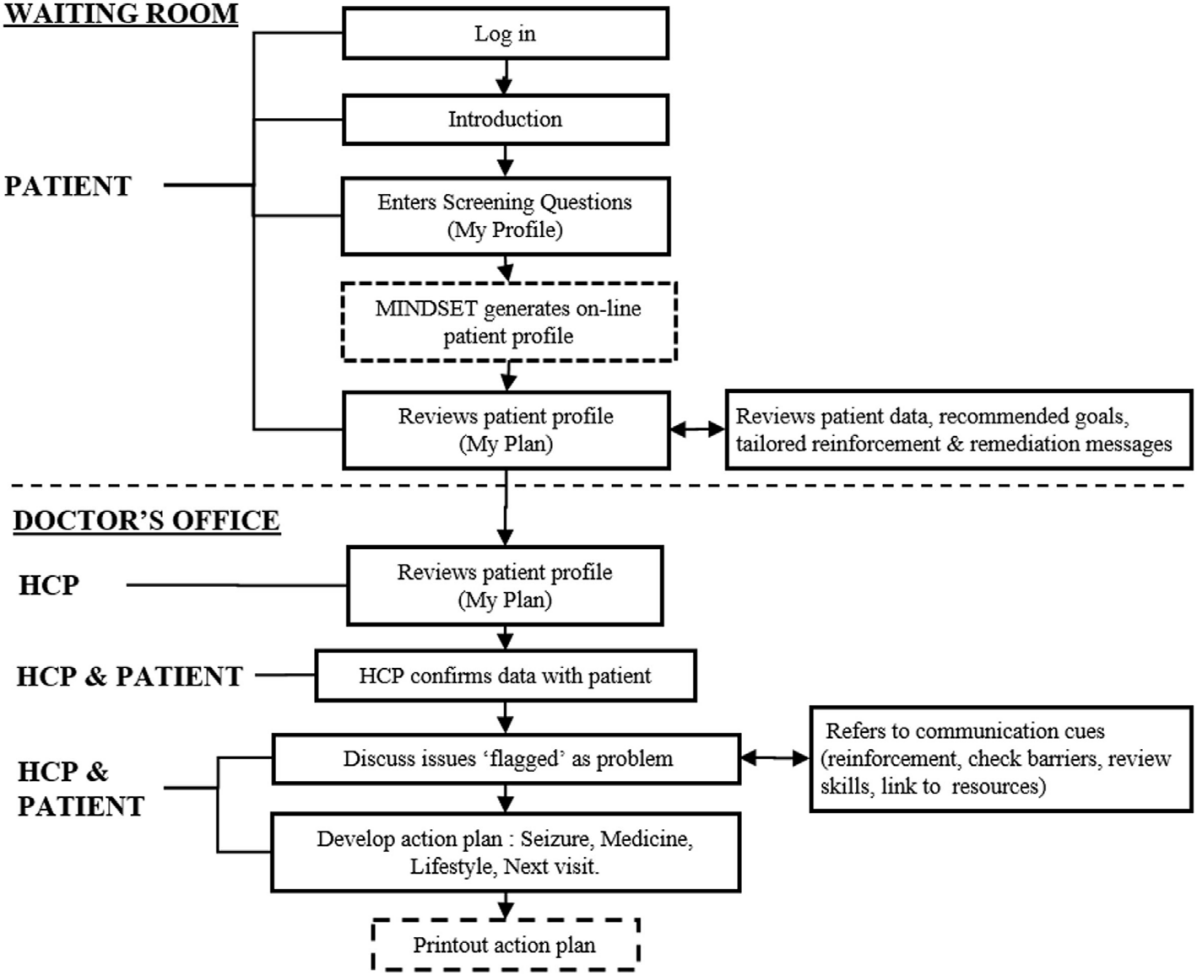
Management Information Decision Support Epilepsy Tool (MINDSET) upper-level flow (101).

Guidance on how MINDSET could align to existing guidelines, recommendations, and clinic flow was informed by the 5 A’s model, quality assurance guidelines, and clinic task analysis. *The 5 A’s Behavior Change Model*. The 5 A’s Behavior Change Model (used with the Improving Chronic Illness Care Chronic Care Model) provided a framework for developing the scope, contextual fit, and application of MINDSET at the interpersonal (patient-provider interaction) level (81). A tenet of the model is that chronic illness patients have a S-M Action Plan covering the 5 A’s elements (Assess, Advise, Agree, Assist, and Arrange). *Quality-of-Care Measures*. Quality-of-care measures for epilepsy management include an array of assessment, treatment, and counseling protocols representing the best practice recommendations (33, 34, 100). Published quality care measures for the clinical management of epilepsy were consulted to determine the context of use for the practice of medicine. Aligning MINDSET function within these protocols positioned it for ready acceptance for clinic use. *Clinic Task Analysis*. Task analysis was conducted to examine the clinic flow in each of the participating clinics to understand the on-site operation and to determine logical opportunities for intervention without compromising that clinic flow (101) (Figures 3 and 4). This involved shadowing patients through their clinic visit in each of the participating neurology clinics, examining data flow within the clinic for each patient, decision-making by HCP, interaction points between the patient and provider, and duration in each location.

##### Task 3.3 Select or Design Practical Applications to Deliver Change Methods

The planning team selected specific practical applications to operationalize the theory-based change methods in ways that fit the population and setting for the intervention. We designed MINDSET to be easy to use by physician and patient and portable to be able to accompany the patient through the clinic visit. A PC tablet-based tailored self-assessment approach appeared feasible for intervention delivery. Inclusion of data familiar and important to HCPs (e.g., seizure frequency and history, medication missed doses, and side effects) were included with the less familiar data on S-M behaviors for seizure, medication, and lifestyle management to provide added salience for use in the clinic setting. Clinic visit time constraints further suggested the advantages of tailoring data input such that patients would only enter their perceived self-efficacy and importance for “flagged” S-M problem behaviors. An action plan that could be printed in the clinic provided a vehicle for use by both patient and provider during the clinic visit as well as an ongoing reference by the patient between clinic visits.

#### IM Step 4: Program Production

Step 4 comprised refinement of the program’s structure and organization, planning for program materials, drafting of messages and materials, and pretesting, refinement, and production of materials.

##### Task 4.1 Refine Program Structure and Organization

Management Information Decision Support Epilepsy Tool is provided on a tablet-based platform to provide S-M decision support to patients (≥18 years) and HCPs during their clinic visit and a printable action plan to provide decision support to patients outside the clinic (102). Originally mounted on an Archos 101 Android tablet platform (and subsequently on a Windows-based Dell platform), the use of MINDSET in the clinic comprises: (1) data entry by the patient; (2) data review by the patient and HCP; and (3) discussion by the patient and HCP of issues, goals, and strategies in conjunction with a tailored action plan (102). MINDSET was designed for the patient to enter data in the waiting room, prior to seeing their HCP. Data represented three epilepsy S-M domains: medication; seizures; and lifestyle. The method of chunking (Table 4, #1) informed us in distilling the complexity of epilepsy S-M into questions assessing 3 management domains and 13 S-M sub-domains including medication S-M (“current AED prescriptions, medication adherence, adherence barriers, side effects, and medication, S-M behaviors”), seizure S-M (“the patient’s recent seizure history, including frequency and type, and seizure S-M behaviors”), and lifestyle S-M (“including mood, social life including sexual relationships, child care, employment, and driving, physical activity, safety, record keeping, social support, and clinic visits”) (102).

**Table 4:** Examples of theoretical methods and practical applications used in MINDSET.

#	Method	Definition	Practical applications
1	Chunking	Using stimulus patterns that may be made up of parts but that one perceives as a whole	Organization of the complexity of epilepsy S-M into sub-categories and domains. For example, the patient completes MINDSET S-M assessment by addressing behaviors related to seizure management, then medication management, then lifestyle management
2	Self-assessment, Consciousness raising, Information transfer	Providing information, feedback, or confrontation about the causes, consequences, and alternatives for a problem or a problem behavior	Providing the patient with an epilepsy S-M profile raises awareness of issues that had previously been ignored. Tailored advice messages on the printed action plan list examples of behavioral strategies to meet S-M goals (see Table 7)
3	Feedback	Giving information regarding the extent to which the individual is accomplishing learning or performance, and the extent to which the performance is having an impact	The patient’s action plan provides an indicator for how a “risk” behavior has changed since the last visit (improved, worsened, no change)
4	Tailoring	Matching the intervention and components to previously measured characteristics of the participant	The patient profile and action plan are tailored on the S-M data provided by the patient. S-M goals are prioritized by flagged behaviors and patient ratings of self-efficacy and importance of the behavior. The patient’s action plan is dynamically built in response to the patient’s individual profile data
5	Reinforcement	Linking the behavior to any consequence that increases the behavior’s rate, frequency and probability	The profile and action plan provide reinforcement messages to patients who have no flagged behaviors through congratulatory statements in the action plan
6	Advance organizing	Presenting an overview of the material that enables a learner to activate relevant schemas so that new material can be associated	The Action Plan delivers a S-M profile and goals in a logical format that mirrors the conceptualization of S-M within 3 domains to simplify understanding of what needs to be done. The MINDSET action plan provides recommended strategies to support S-M goals to prevent seizures
7	Goal setting	Prompting planning what the person will do, including a definition of goal-directed behaviors that result in the target behavior	Commitment to S-M goals that are agreed on by patient and provider. Flagged behaviors are reframed in the action plan as S-M goals (e.g., Make sure to get enough sleep)
8	Cues to action	Providing opportunities for learners to have personal questions answered or instructions paced according to their individual progress	Cues are provided for the patient to discuss “at-risk” (flagged) behaviors with the HCP during the clinic visit
9	Planning coping responses	Getting a person to identify potential barriers and ways to overcome these	Discussion of causes for non-adherence of anti-seizures medication and review of recommended strategies to derive ways to overcome barriers to adherence
10	Cue altering	Teaching people to change a stimulus that elicits or signals a behavior	A strategy is provided to introduce cues to pack sufficient anti-seizure when packing for a trip
11	Self-monitoring	Prompting the person to keep a record of specified behaviors	Recommended strategies for monitoring include record keeping (e.g., a symptom diary and seizure tracking) to enable better understanding of seizure antecedents
12	Facilitation/Linkage to care/support	Creating an environment that makes the action easier or reduces barriers to action	MINDSET provides linkage to community resources and support groups that are also printed in the action plan (e.g., Epilepsy Foundation)
13	Repeated exposure	Making a stimulus repeatedly accessible to the individual’s sensory receptors	MINDSET is provided at each clinic visit

###### Patient Data Entry for Assessment

**Figure 5:**
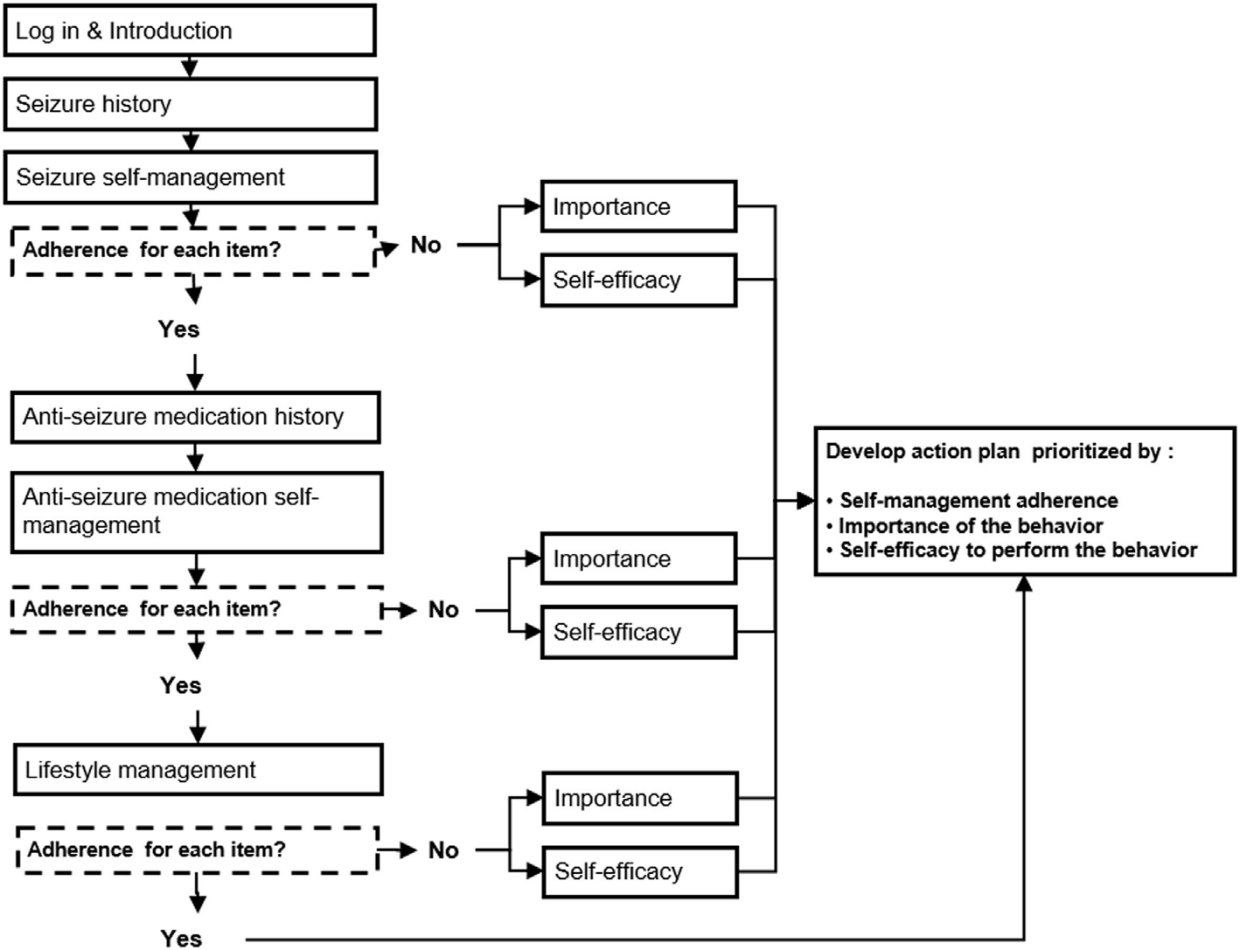
Management Information Decision Support Epilepsy Tool (MINDSET) decision flow to produce a tailored action plan (101).

Scales were embedded in MINDSET to provide assessment of the critical behaviors and determinants previously identified (Tables 1 and 2). A design specification was that MINDSET be minimally intrusive of clinic flow and patient burden. Therefore, an assessment battery was designed that collected information based on theory and empirical relevance, availability of a comprehensive and psychometrically valid scale, and clinical practice needs. For this reason, the determinant of knowledge was not assessed in MINDSET, though addressed in tailored messaging and action plan feedback. Furthermore, in response to the need for utility for use, assessment was tailored such that data were collected only when necessary for a given patient. For example, data on perceived self-efficacy and importance were only collected on a behavior if that behavior was flagged as “at-risk” (less than optimal adherence frequency), described in Task 4.2 and Figure 5 below.

###### Assessment of S-M Behaviors

Self-assessment was an important method applied within MINDSET (Table 4). Assessment of S-M behavior was collected using the 38-item *Epilepsy S-M Scale* (7, 8, 16, 55) that delineates behaviors regarding medication adherence, seizures, information, safety, and lifestyle. Responses were entered using a button selection on a 5-point Likert scale ranging from “never” to “always.” Perceived self-efficacy to perform S-M behaviors was assessed using a 33-item *Epilepsy Self-Efficacy Scale* (7). Responses were entered on a sliding scale (slider bar) adapted from motivational enhancement protocols (86) with a response set ranging from 0 to 10 with 0 being not at all confident (I cannot do at all) and 10 being extremely confident (Sure I can do) (8, 56). Self-efficacy items were completed for those behaviors flagged as “at-risk.” Also adapted from the use of decision rulers from motivational enhancement protocols was the assessment of importance. Responses were based on a sliding scale from 1 to 10 with 1 indicating not important and 10 indicating extremely important (86).

###### Assessment of Medication Side Effects and Barriers to Adherence

Medication side effects represent an important clinical parameter to inform AED prescription as well as motivation for medication adherence. Side effects were assessed using a 19-item *Epilepsy Adverse Events* profile assessing reported problems during the previous four weeks from a list of 19 adverse effects (Table 5) (34, 103–105). The scale assessed reported problems during the previous 4 weeks from a list of 19 adverse drug effects (Table 5). The original instrument used a 4-point Likert scale response set: 1. Never a problem; 2. Rarely a problem; 3. Sometimes a problem; 4. Always a problem (103). Barriers to AEDs were assessed using a list of 18 barriers to medication adherence (adapted from previous studies) and provided to patients reporting missed doses (Table 5) (88).

**Table 5:** Items assessing medication side effects and barriers.

Anti-seizure medicine side effects (adverse effects scale)		Medication barriers (adapted from HIV scale)
None Unsteadiness Tiredness Restlessness Aggression Nervousness Hair loss Skin changes or rash Blurred vision Upset stomach	Concentration difficulty Mouth/gum problems Shaky hands Weight gain Dizziness Sleepiness Depression Memory problems Disturbed sleep	I simply forgot I don’t like taking pills I thought the drug was toxic or harmful I felt depressed or overwhelmed I felt sick I wanted to avoid side effects I was away from home I was busy with other things I had a change in my daily routine I found it difficult to take pills at specified times I slept through the dose time I did not want others to notice me taking medication I had too many pills to take I ran out of medicine and didn’t fill the prescription in time I have difficulty storing/carrying meds I have difficulty paying for meds I have problems filling my prescription Other

###### Assessment of Depression

Depression is a common co-morbidity of epilepsy that can compromise S-M practice. MINDSET was not initially designed to intervene on depression directly and S-M matrices were developed for patients who were physically and cognitively capable of S-M practice. However, the MINDSET planning team saw the potential of MINDSET providing neurologists with the benefit of rapid assessment. Depression was assessed using the 6-item Neurological Disorders Depression Inventory for Epilepsy (NDDI-E) Screening Tool that assesses the degree of depressive symptoms in the last week (106–108). Patients were “prompted to provide the answer that best described them over the last 2 weeks for ‘everything is a struggle’, ‘nothing I do is right’, ‘I feel guilty’, ‘I’d be better off dead’, ‘I feel frustrated’, and ‘I had difficulty finding pleasure’. The response set was a 4-point Likert scale ranging from never to always or often (102).” “NIDDI-E scores of above 15 were considered positive for depression, with specificity of 90%, sensitivity of 81%, and positive predictive value of 0.62” based on the mini international neuropsychiatric interview (MINI) (102, 107, 108).

###### Patient Review of the S-M Profile

Immediate feedback is an important method applied in MINDSET (Table 4, #3). A profile is produced by MINDSET. The patient can review this in the waiting area and then share it with the HCP (Figure 3). The profile summarizes responses on medication, seizures, and lifestyle, and flags “at-risk” behaviors based on a comparison of the frequency of the behavior to benchmarks. As previously described, the patient rates his/her self-efficacy (confidence) and perceived importance to perform any S-M behavior that is flagged (Table 4, #4). Based on programmed benchmarks for behavior (frequency), self-efficacy (degree of confidence), and perceived importance, the profile provides a prioritized list of behavioral issues for discussion, goal setting, and action. The profile has accompanying tailored advice boxes to increase awareness about strategies to improve S-M behaviors. If the patient reports no problems with S-M behaviors (i.e., he/she has no flagged behaviors), reinforcement is provided in a text-based congratulatory message (Table 4, #5). The advice boxes are also available to provide anticipatory guidance (or advance organizers) in the form of specific behavioral strategies to consider in the future (Table 4, #6). When sharing MINDSET, both the patient and HCP can tab to a list of recommended action items and discuss the items and set goals (Table 4, #7). “The process of using MINDSET is designed to promote shared decision-making where a patient and HCP can assess the need for improvements (both medical and psychosocial) and make subsequent informed treatment and behavioral change decisions (102).” The applications, messages, and cues for discussion (Table 4, #8) are designed to impact determinants of knowledge, self-efficacy, perceived importance, and skills (Table 1).

###### HCP-Patient Review and Discussion of the S-M Profile and Action Plan

Providing patients with a decision aid to document S-M behaviors and guide future S-M goals is consistent with other approaches to chronic disease management (e.g., asthma) (109). For epilepsy S-M, such tools have focused on acute seizure management and not broader S-M domains inclusive of medication or lifestyle behaviors (110). MINDSET flow and function provides the HCP with an intuitive scaffold to progress through the management steps of assess, advise, agree, assist, and arrange (Table 2), allowing a rapid review of a patient’s status, reviewing strategies to plan coping responses (Table 4, #9), to alter behavioral cues (Table 4, #10), to institute self-monitoring (Table 4, #11), and to link to family and community support as needed (Table 4, #12). The process of using MINDSET is reiterated at each clinic visit (Table 4, #13).

##### Task 4.2 Prepare Plans for Program Materials

A program design document provided the blueprint for MINDSET, informed by our understanding of patient characteristics, including knowledge, education, cognitive capacity, and time available for learning and discussion in the clinic setting (10). The team developed flowcharts to establish the function of MINDSET for the programmer, depicting the steps in the development of a tailored S-M action plan focused on AED adherence, seizure management, and lifestyle management (Figure 5). Flow charts and screen map mock-ups were developed as Powerpoint slides to depict MINDSET content, function, position of menu options, data entry components, patient profile display screens, and tailored feedback (bullets and cues). These “proof-of-concept” layouts illustrated what the patient and provider would see (the look and feel of the program).

**Figure 6:**
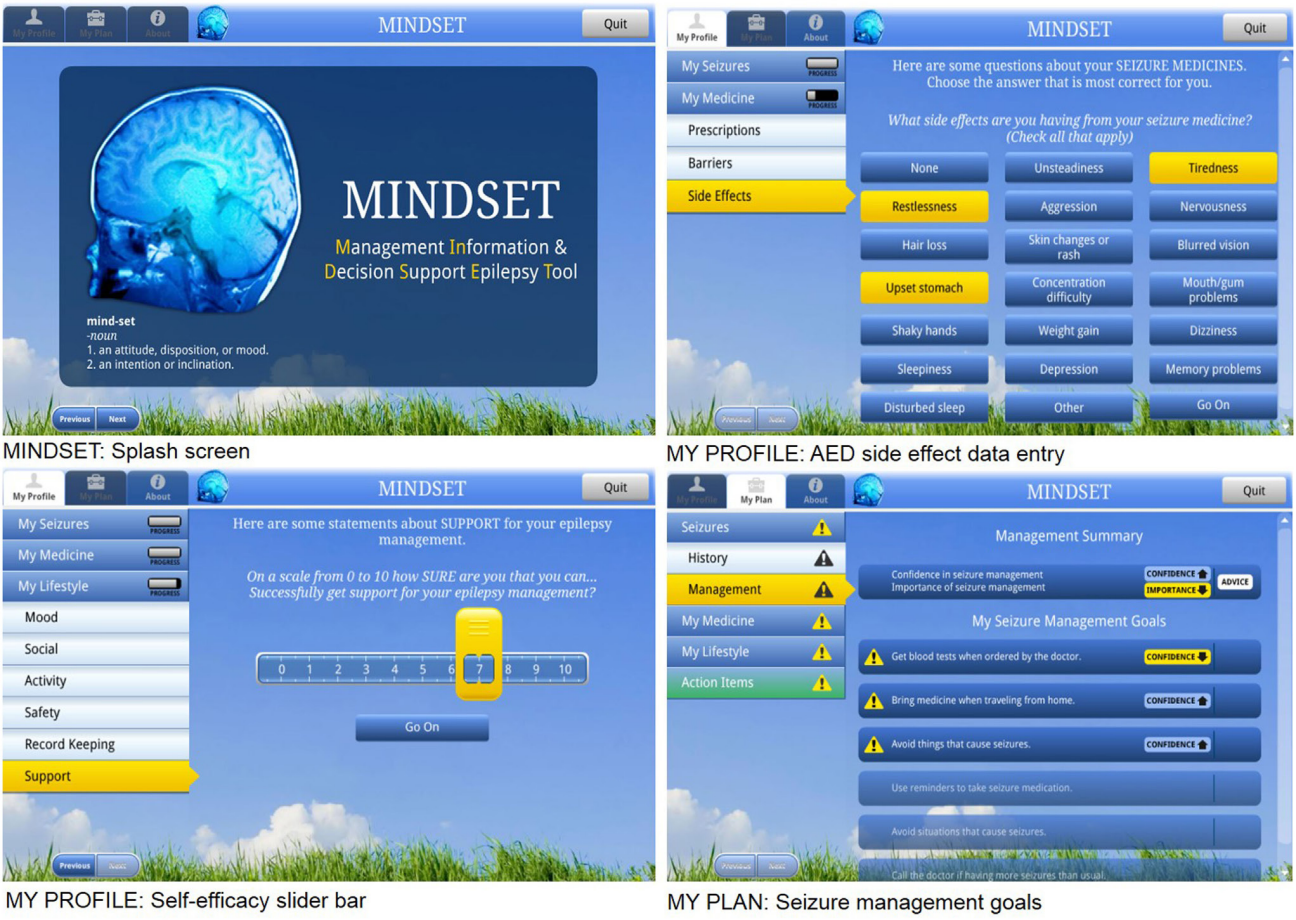
Management Information Decision Support Epilepsy Tool (MINDSET) user interface (101).

Initial mock-ups depicted the following: (1) screening assessment and (2) decision support for intervention on S-M (Figure 6). The screening assessment consisted of computer-based prompts for the patient to input data (based on the data acquired from the screening tool, see Section “Task 4.1 Refine Program Structure and Organization” above) (10). The decision support was designed to provide feedback to both the patient and the provider in the form of confirmation of the patient’s profile on clinical and psychosocial variables; cues on discussion points during the clinic visit; and S-M goals and an action plan for after the clinic visit. The algorithm for prioritizing the S-M goals on the basis of patient self-report is illustrated in Figure 6. The development of flowcharts and screen maps was an iterative process and an essential one that helped guard against serious error or logical flows in the finished product. The design of an intuitive user interface was essential so that someone unfamiliar with the program could easily use it. A dedicated formative PPAG meeting, held at the KS Clinic conference room, provided a review and feedback on the design documents including content, design (interface) features, navigation, functionality, language, logistics of use and implementation in the clinic, orientation needs, and evaluation specifications. The aim was to uncover any concerns with these program elements as well as recommendations for improvement of MINDSET for PWE prior to programming. The PPAG was provided a simulated “walk through” of MINDSET from log-in through action plan review using projected screen “mock-ups” on Powerpoint slides. Flowcharts were used to illustrate MINDSET use in the context of the clinic visit. The PPAG had few concerns about the use of MINDSET within the clinics and the top-level flow of the program. Their concerns were mainly focused on clarity and completeness. Suggestions for improved clarify included defining medical terms (e.g., in describing seizure types) and specifying general terms (e.g., “wellness”). Suggestions for completeness included adding “choose all that apply” and “I don’t know” options to data collection items; adding dosage amounts for assessment of medication adherence; and addition of items focused on negotiating independence and privacy. Design document revisions were made in response to PPAG consensus.

##### Task 4.3 Draft Messages, Materials, and Protocols

Programming followed a stepped sequence. At each developmental step all components of the program were taken one draft further toward completion, building upon the review of previous developmental steps. This process ensured that all elements of the program had been developed with the benefit of multiple reviews by the research team. Structured programming techniques were used to develop the program and reduce needed refinements. The Archos 101 Android tablet platform provided the first MINDSET hardware platform, later superseded by the Dell Latitude. Patients and providers interacted with MINDSET using a stylus or touch screen. The program was button and menu driven and designed for intuitive, easy navigation for both patient and provider with a limited depth of screens, ensuring providers could review the patient’s entire profile in two stylus button presses and not need to “drill down” for data deeper than two screens.

Data input was in the form of pre-existing items from the previously validated surveys embedded in MINDSET (see Task 4.1 Refine Program Structure and Organization above). Tailored messages were created from permutations of these data. Segue messages confirmed the patient’s self-efficacy (low/high) and perceived importance (low/high) regarding a particular flagged behavior and provided a cue to the need for further discussion and reference to the action plan (Table 6). Tailored messages in the action plan were guided by the 5-A’s model: Confirming the patient’s S-M profile (including citing the flagged behavior) and reinforcing S-M success (Assess), stating the behavior goal to mitigate the behavioral problem (Agree), providing strategies and recommendations specific to flagged behaviors (Assist) and cued discussion with the HCP (Advise) (Table 7).

**Table 6:** Tailored Segue Messages Based on Confidence and Importance Feedback Exemplified for Medication Management.

Self-efficacy	Importance		HCP POs (Table 2)
	Low	High	
Low	You have reported that you are not confident that you can take your seizure medicine as your doctor has prescribed and don’t think this is highly important to do	Congratulations on recognizing the importance of taking your seizure medicine as your doctor has prescribed. Despite this, your answers suggest that you are not confident of taking your medicine as your doctor prescribed	PO.1.iii. Assess patient attitudes (importance and confidence) regarding S-M behaviors PO.1.iv. Provide patient with personalized feedback on epilepsy status and S-M for review
	*Advice*: Discuss this with your doctor and use the activities listed in your MINDSET action plan to help you	*Advice*: Discuss this with your doctor and use the activities listed in your MINDSET action plan to help you	
High	Congratulations on being confident that you can take your medicine as your doctor prescribed. Despite this, your answers suggest that you don’t think taking medicine is highly important	Congratulations on recognizing the importance of taking your medicine and being confident that you can follow your prescription plan	
	*Advice*: Discuss this with your doctor and use the activities listed in your MINDSET action plan to help you	*Advice*: Use the MINDSET program to help you and your doctor provider review all the aspects of your epilepsy S-M	

**Table 7:** Example Management Information Decision Support Epilepsy Tool (MINDSET) messaging and associated objectives for a patient who reports forgetting to take seizure medicine.

5-A steps	Message	Associated objectives
*Assess*: confirm status	*What you told MINDSET*: you [sometimes, always] forget to take your seizure medicine. You should feel proud of all the times you have taken your seizure medicine as your doctor has prescribed. Forgetting to take your medicine can cause seizures so make sure you talk to your doctor about this	*Change objective (HCP* Table 2*)* PO.2.iii. Provide specific, documented behavior change advice (action plan) in the form of a prescription

*Agree*: make this part of your goal	*Your goal*: Make reminders to take your seizure medicine part of your daily activities	

*Assist*: develop strategies to overcome barriers, refer to evidence-based education, refer to resources, discuss with your HCP	*Your strategy*: Try these actions if you have problems remembering to take your seizure medicine: Take your medicine with daily activities (breakfast, dinner, during TV show, before going to bed) Use a pill container Use a calendar or a set a daily reminder on your phone’s calendar Use a seizure diary to keep track of when you take medicine Use electronic reminders, text or email, sent to you when it’s time for your medicine. See “My Epilepsy Diary” or “Texting 4 Control” in the resource list of your action plan.	*Change objective (Patient* Table 1*)* K2iii. List cues to action (memory aids) for taking meds correctly (e.g., by toothbrush, pill box, at mealtimes)

*Advise*: cue discussion to acknowledge, reinforce, and agree on strategies to meet S-M goals	Patient and HCP are cued to discuss this “flagged” behavior to: (1) acknowledge status (2) reinforce past successes (3) reach agreement on the goal (4) review and agree on strategies (5) review barriers to the selected strategies and how to overcome these	*Change objectives (Patient* Table 1*)* S2iii. Demonstrate how to use cues/memory aids SE2iii. Express confidence in ability to use cues/memory aids *POs (HCP* Table 2*)* PO.4.i. Help patient develop strategies to address barriers to change (write on Action Plan form) (ask is there anything that would prevent you from doing these strategies) PO.4.ii. Refer patient to evidence-based education or behavioral counseling – individual or group PO.4.iii. Elicit patient’s views and plans regarding potential resources and support within family and community

*Arrange*: printout and linkage	Messages printed in the action plan including community resources	*Associated change objectives (Patient* Table 1*)* K2iii. List cues to action (memory aids) for taking meds correctly (e.g., by toothbrush, pill box, at mealtimes) Associated POs (HCP Table 2) PO.5. Provide the patient their personalized S-M Action Plan and follow-up call to patient within a week after visit as “booster” for S-M Action Plan PO.6. Link patients to clinical and community resources appropriate to support and resource needed

##### Task 4.4 Pretest, Refine, and Produce Materials

Upon completion MINDSET was pretested and refined through an in-house alpha test and a usability test.

###### MINDSET Alpha Test for Functionality

An in-house alpha test was conducted by the MINDSET research team to ensure all program components and functions conformed to the intentions of the designers, functioned appropriately, and presented no anomalies (“bugs”). Research team members each completed MINDSET, simulating a patient with particular epilepsy S-M profiles. They completed logs recording any problems encountered that included incorrect logic, program bugs, syntax errors, or interface design problems. They completed the problem log by recording their location in the program, the user initiated events that preceded the problem, and a description of the problem (including screen captures where appropriate). Problems were collated and sent to the programming team for further troubleshooting and revision prior to usability testing.

###### MINDSET Usability Testing with PPAG

Patient Provider Advisory Group patients from three clinic sites (36–53 years of age, prescribed at least one AED, mainly female and ethnic minority) and 4 HCP from the planning team were asked to use the MINDSET prototype in “laboratory” conditions (a dedicated conference room at Kelsey–Seybold clinic) not associated with their regular clinic visit. Hypotheses for usability testing were that patient ratings on usability parameters (measured on a usability survey) would exceed an *a priori* benchmark of 70% agreement and that HCPs would rate MINDSET features (measured on a features checklist) as providing improvement to their current practice. After an orientation, patients were asked to access all elements of MINDSET (the screening tool, patient profile, recommendations, and action plan) and to verbally describe and interpret what they were seeing and doing. Any problems (as previously described for alpha testing) were recorded and collated. Patients then completed a usability survey assessing the functions of MINDSET and were interviewed on how MINDSET could be improved in terms of content, function, and interface design. Data were gathered on the patient’s satisfaction with the user interface, ease of use (usability), acceptability, credibility, and applicability of the system to their needs using previously validated usability measures. HCPs were provided with a MINDSET tablet that had pre-loaded data on a patient whose profile indicated clinical and psychosocial S-M needs. Patients rated MINDSET favorably on usability parameters, providing 80 to 100% agreement that it was easy to use, likable, credible, understandable, and appealing. This exceeded *a priori* success criteria of at least 70% agreement (94, 95). Patients appreciated the opportunity to thoroughly review their epilepsy management: “It makes me look @ problems in my lifestyle/mood,” and to receive advice: “I *love* the advice sections”; “the advice sections were really useful for me”; and to organize their thoughts prior to the clinic encounter “:… opportunity to remember everything to discuss with doctor”; “the information and seizure history for the doctor is great”; and “Helped condense my thought and organized any questions I might have.” HCPs rated MINDSET as increasing the ease, thoroughness, accuracy, and communication in each of the S-M domains (“seizure history and management, medication management, lifestyle management, and providing an epilepsy action plan”) (102).

Reported barriers to use of MINDSET included that the questions (behavior and self-efficacy) seemed repetitive; that patients required assistance due to technical difficulties with the tablet that delayed system responsiveness (distinct from a need to clarify data input questions); and that, while patients advocated the use of MINDSET, they suggested the need for patience for data entry due to the extensive data input in the My Profile section. Modifications were made in response to these issues. These focused on technical/functional fixes, on adjusting clinic expectations on the time commitment for data entry, and alerting patients to the apparent repetition of data input items. The usability data indicated that MINDSET showed initial promise in facilitating the operationalization of S-M constructs for screening, management, and education; the application of clinical guidelines; and was feasible for clinic use. The HCPs rated MINDSET favorably on thoroughness; but also rated it as requiring more time for the clinic encounter.

#### IM Step 5: Implementation Plan

Step 5 comprised describing potential program implementers, stating the outcomes and POs for implementation, constructing matrices of change objectives for implementation, and designing implementation interventions. An implementation intervention for wide scale adoption, implementation, and maintenance of MINDSET can be developed pending the intervention’s demonstrated efficacy to enhance epilepsy S-M behaviors.

##### Task 5.1 Identify Potential Program Implementers

Management Information Decision Support Epilepsy Tool was designed for use by HCPs in specialty neurology clinics managing outpatients with epilepsy. Thus, potential adopters included specialty clinic directors or upper-level administrators; potential implementers included HCPs such as neurologists, epileptologists, and nurse educators.

##### Task 5.2 State Outcomes and POs for Implementation

Performance objectives for adoption were brainstormed by the research team with consideration of the decision-makers in neurology clinics, and informed by the IM framework (38), and characteristics for diffusion of innovation (111). These included that implementers recognize a need for MINDSET and its relative advantage, and make a formal commitment to use. Steps drafted to date include that the clinic director will: Assess the need for an epilepsy S-M program among clinic patients; review MINDSET and note objectives and relative advantages for program adoption; obtain feedback from clinic staff on potential barriers to/advantages of adopting MINDSET; solicit experiences from other clinics that have used MINDSET; agree to adopt MINDSET by signing a memorandum of understanding for its use.

##### Task 5.3 Construct Matrices of Change Objectives for Implementation and Task 5.4 Design Implementation Interventions

Critical opportunities for MINDSET implementation within the clinic flow were identified from clinic task analysis of collaborating clinics. This enabled us to understand environmental constraints. MINDSET was designed to accommodate regular clinic visits in varied clinic settings previously described (Figures 3 and 4). Matrices of change objectives for clinic directors, HCPs, and clinic nurses to adopt and implement MINDSET and the development of an implementation intervention are pending determination of its effectiveness.

Management Information Decision Support Epilepsy Tool will be more likely adopted if it is efficacious with minimal disruption to clinic activities or clinic overhead. The thoroughness of the S-M assessment may be associated with greater time commitments but this may, in turn, be offset by its provision of a detailed record of (potentially) billable behavioral counseling activities in the clinic. Integration of the MINDSET data base with existing medical record systems would also enhance its appeal to HCPs. Emerging potential uses for MINDSET exist beyond its original design including as a tool for clinic-based community health workers and as an electronic behavioral assessment with the National Epilepsy Education and Awareness Collaborative (NEEAC).

#### IM Step 6: Evaluation Plan

Step 6 comprised effect and process evaluation questions, developing indicators and measures of assessment, and specifying an evaluation design.

##### Task 6.1 Write Effect and Process Evaluation Questions

The primary question to be addressed in planning the evaluation of MINDSET was: Does the use of MINDSET by a PWE and their HCP during multiple clinic visits over a 9 month period, including the use of a printed action plan between visits, improve the S-M behaviors and confidence of patients? Stated as an alternative testable empirical hypothesis: PWE who use MINDSET in the context of their usual clinic visit for three consecutive clinic visits over a 9-month period, and a printed action plan between visits, will report at least three fewer “at-risk” S-M behaviors (assessed by the Epilepsy S-M scale) compared to patients who do not use MINDSET.

Planned process evaluation questions included assessment of factors that mediate the success of MINDSET as well as facilitating its implementation (Table 8). These include intervention exposure, impact on patient-provider communication, and information seeking other than MINDSET. Sufficient exposure to MINDSET relates to implementation fidelity, that the patient was exposed to all components and completed them through to action plan printout. Incomplete exposure compromises the quality of the efficacy trial. Time-on-task data (both patient and patient and provider use) informs expectations for future implementation (e.g., time commitments) for adopting clinics. Assessment of the quality of the patient and provider clinic encounter when MINDSET is used allows a determination of correspondence between MINDSET cues and topics subsequently discussed in the clinic encounter. Exit interviews allow for a protracted discussion of the HCP’s experience in using MINDSET and recommended adjustments to facilitate its use and future adoption. Assessment of the degree to which the patients accessed other sources for information on S-M enables an accurate assessment of the degree to which MINDSET and the action plan (distinct from other sources) influenced S-M. Knowledge gained from clinic testing will inform implementation plans and program user manuals for those adopting MINDSET in the future.

**Table 8:** Sample measures for pilot test.

#	Instrument (impact)	Description
**Self-management (S-M) behavior**		

1	Epilepsy S-M Scale	38 Likert scale items. Internal consistency (alpha) = 0.81–0.84. Principal components analysis with varimax rotation yielded 5 factors (7, 8, 16, 54)

**Self-efficacy**		

2	Epilepsy Self-efficacy Scale	Consists of 33 items using an 11 point rating scale, ranging from 0 (I cannot do at all) to 10 (Sure I can do). Items yield a total summative score. Content and construct validity have been assessed in a 25 item version of this scale with alpha coefficients ranging from 0.91 to 0.94 (8, 16, 54)

**Depression**		

3	Neurological Disorders Depression Inventory for Epilepsy (NDDI-E)	The scale is well validated, has high internal consistency (alpha = 0.80), test-retest reliability = 0.78 (106–108)

		**Process measures**

4	Intervention exposure	*Aim*: To monitor the extent of implementation and the degree and fidelity of MINDSET delivery. *Measure*: Data collected within MINDSET on selections and preferences made within the program and time-on-task.

5	Clinic encounter	*Aim*: To understand if the application of MINDSET influences patient-provider communication during the clinic visit. *Measure*: The patient will complete a clinic visit interaction checklist, clinic visit communication scale, and shared decision-making scale immediately after their clinic visit to assess quality of communication.

6	Exit interview	*Aim*: To understand the patient’s and HCP’s experiences with MINDSET, the most useful components and features, barriers to use, suggestions for improvement, and ratings on the MINDSET’s perceived impact on epilepsy management. *Measure*: Patients and HCPs will complete an exit interview adapted from previously reported protocols.

7	Information seeking behaviors	*Aim*: Information seeking is an activity that may occur following completion of the pretest evaluation survey items (all patients) or in response to MINDSET (treatment patients) and can be an important mediating variable. *Measure*: Participants will be asked whether they actively sought information about epilepsy and where they looked for this information, e.g., Internet, Foundations, medical practitioners, or popular media sources (radio, television, or newspapers).

##### Task 6.2 Develop Indicators and Measures for Assessment

From the outset the development of MINDSET focused on instruments and scales to assess patient’s S-M status and to provide indicators of S-M success over time. For this reason, measures for evaluation can closely correspond to those embedded in MINDSET. *Planned impact measures* include the epilepsy S-M scale, epilepsy self-efficacy scale, NDDI-E, and adverse effects scale previous described (refer to Step 4: Program Production and Table 8). *Planned process measures* were developed to assess the process evaluation constructs previously described (Table 8).

##### Task 6.3 Specify Evaluation Design

The planned evaluation design for MINDSET involves an RCT with a sample of patients randomly assigned to treatment (MINDSET and usual care) and comparison (usual care only) groups (*n* = 30 per group) at three clinic sites over three visits to evaluate its efficacy.

###### Planned Patient Recruitment

A total of 60 patients from the KS clinic (*n* = 20), BT clinic (*n* = 20), and UT clinic (*n* = 20) (previously described) would be invited to participate. Participants would include patients with a diagnosis of epilepsy who are 18 years of age and older, who can speak English, who are willing and able to complete MINDSET, and who have no other medical disorders that could inhibit their ability to use MINDSET or practice S-M activities. Participation would be based on clinician and nurse educator referral and ideally reflect the diversity of gender and race-ethnicity, average age and SES for the respective clinic populations.

###### Planned Pilot Efficacy Trial of MINDSET

Each clinical site would recruit 20 patients to participate in the randomized pre–post treatment and comparison group study. Once enrolled the patients would participate during three regular clinic visits that would be scheduled three months apart. They would be randomly assigned to one of two groups (30 in each group, 10 from each site) for receipt of the treatment (MINDSET plus usual care) or comparison (usual care only) condition.

At the *first visit*, a MINDSET research staff member would meet the patient at the clinic to confirm participation, answer questions, and, if they agree to participate, obtain signed consents. Consent and study protocols are subject to approval by human subjects internal review boards at the contributing university and clinical organizations. All patients would then complete a contact sheet, and a demographic survey. They would then input data into the assessment section of MINDSET (My Profile) prompted by screening questions. This would include data on seizures, AEDs, and lifestyle, as well as S-M behaviors (Epilepsy S-M Scale) and self-efficacy (Epilepsy Self-efficacy Scale) related to these domains. Data input would take place in the waiting room and clinic room while waiting for the clinic appointment.

Group 1 patients would use MINDSET to review their epilepsy S-M patient profile (My Plan) that indicates S-M challenges (“risk”), provides behavioral goals and associated advice about content, provides recommendations for discussion with the HCP, and also provides information on associated S-M resources (e.g., available through the American Epilepsy Society, Epilepsy Foundation, and MEW Network). During the clinic encounter, the provider and patient would refer to MINDSET. The HCP would be provided suggested action items based on the patient’s profile, could access the patient profile (My Plan) data and could confirm or modify these data after interviewing the patient. The patient and provider would have the opportunity to review recommended discussion points, goals for management, and the action plan. The provider will have the opportunity to provide the patient with a tailored printed action plan that reiterates the priority management goals discussed in the clinic encounter.

After completing initial assessment items in MINDSET, Group 2 patients would provide MINDSET back to the research staff member and continue their regular clinic visit in which they would meet with their providers as usual without the benefit of MINDSET information and prompting on discussion points and the action plan, and without the receipt of a printout of the action plan. Following the clinic visit both group 1 and group 2 patients would complete process measures of the clinic visit interaction checklist and clinic visit communication scale. All patients will then be provided $15 for their participation.

During the *second visit*, patients in Group 1 and Group 2 would again complete the assessment (My Profile) component of MINDSET. Group 1 patients would again use MINDSET to review their epilepsy S-M patient profile and recommended management goals (My Plan) and both HCP and the patient can use MINDSET to review and confirm data and develop the action plan. The HCP would also have access to any change in the patient data since the last clinic visit. Group 2 patients would again only complete initial assessment items in MINDSET, and then will provide MINDSET back to the research staff member and continue to the regular clinic visit. Again, following the clinic visit, both Group 1 and Group 2 patients would complete process measures (described below). During the *third visit*, both Group 1 and Group 2 patients would input their profile data using the assessment screening tool in MINDSET (My Profile). They will then return MINDSET to the research staff, complete a short exit interview.

###### Analysis

Comparisons of changes in scale scores on S-M and self-efficacy from O_1_ to O_3_ will be made and t-tests and one-way analysis of covariance of the changes will be used to address the evaluation hypothesis. The independent variable of interest in all cases will be group assignment: treatment or comparison. Measures of mediating factors including depression and pre-test scores will be used as covariates.

###### Limitations

This study represents a modest trial designed to be accomplished with available resources. While loss to follow-up is often an issue in such trials the small sample and previous success of the clinics in tacking and maintaining these patients indicates that a 20% attrition is realistic. A per-protocol analysis is planned in this efficacy trial to determine impact of the MINDSET intervention if received. Furthermore, because a limited number of providers (neurologists) are involved in the trial there is expected to be limited turnover and the capability of targeted training by the research staff. Despite this, any findings need to be interpreted in the light of acknowledged study limitations. We expect minimal between-patient contamination because the patients are not typically interacting with each other in these clinics. However, the study is subject to within-provider contamination because a limited number of providers will encounter both MINDSET (Group 1) and usual care (Group 2) patients. It is not possible to blind the provider in this type of trial because they are using MINDSET and the action plan with their patients. It is, therefore, possible that providers will be more attentive to lifestyle issues with all their patients, above what they might originally have been. The contamination will work against a Type I error, making any findings more robust. Further, the time constraints of a busy neurology clinic will likely limit providers to patient-specific cues (from the Action Plan) and not unduly influence general discussion. This remains to be determined. The trial has not been registered in a public trials registry.

## Discussion

Management Information Decision Support Epilepsy Tool is designed to address the need, identified in the 2012 IOM report, “Epilepsy Across The Spectrum,” for substantial engagement by patients and their HCPs to manage therapy and lifestyle issues so as to prevent seizures and maximize quality of life (3, 22, 24). It also provides a called for “structured approach to addressing and documenting patient-centric quality indicators for epilepsy patient care” (3, 22, 24). In epilepsy care, DSSs have focused on diagnostic and pharmacologic support, consistent with historic applications of such systems in medicine (26–31). This reflects the focus and enquiry into the “technical aspects of care, in contrast to personal or social concerns” (32). The development and testing of MINDSET may lead to a new patient-centered decision-making tool to assist in identifying initial steps toward epilepsy S-M (4) to identify patients needing more assistance, and to provide ongoing decision support through action plans. It is also responsive to the Healthy People 2020 objective (HC/HIT-1.1) to increase the proportion of persons receiving easy-to-understand instructions about what to do to take care of their illness or health condition (109). To date, there is a lack of attendance to S-M needs in clinical settings despite the availability of evidence-based interventions to promote epilepsy S-M outside clinic settings.

The IM framework has facility in developing DSSs that promote patient and provider decision-making regarding S-M. Advantages of the framework include the imposition of a systematic approach, thoroughness in detailing needs and solutions informed by theoretical- and empirical literature, encouraging “downstream” thinking regarding implementation, evaluation, and dissemination, and ensuring that priority populations are consulted throughout. Challenges for the use of IM are the time resource required to complete the process with maximum “textbook” fidelity due to the tension with resource constraints in research projects. The MINDSET development presented here represents one case study application for decision support for chronic disease S-M in clinic contexts. In this capacity it is contributive as a guide for future development in analogous domains and populations and application. However, there are necessary limitations in this work that are a basis for future recommendations. Developers are encouraged to apply a systematic application of core processes with each development task. These include posing questions, brainstorming answers, review findings from published research, accessing and using theory, identifying and addressing the need for new research, and formulating the working list of answers. This will ensure a more complete and continuous data feedback loop throughout. Also advised is a timeline that indexes dialog with the priority population with each development task rather than, for example, a calendar meeting schedule which may fail to involve the priority population fully and at the time when input is most helpful.

By providing tools and procedures for identifying and assisting patients with S-M needs, this study will make a significant contribution to the CDC-MEW goal of promoting S-M and self-determination principles in the care of PWE. IM was conducive to providing an innovative technological application to facilitate the dissemination of knowledge from social and behavioral research on epilepsy S-M into clinical practice.

## Ethics Statement

This study was carried out in accordance with the recommendations of local human subject research internal review boards at the University of Texas and the Harris County Hospital District (Harris Health) with written informed consent from all subjects. All subjects gave written informed consent in accordance with the Declaration of Helsinki. The protocol was approved by the local human subject research internal review boards at the University of Texas and the Harris County Hospital District (Harris Health).

## Author Contributions

The authors (RS and CB) have both been involved in the development of the MINDSET epilepsy decision support system from early needs assessment and descriptive studies through the structure and function of the program.

## Conflict of Interest Statement

The authors declare that this research was conducted in the absence of any commercial or financial relationships that could be construed as a potential conflict of interest. The authors and their academic institution have received no payment or services from a third party for any aspect of this research. There are no financial relationships with organizations or entities that could be perceived to influence, or that give the appearance of potentially influencing, what has been written in this work. No commercial patents or copyrights are or have been pending, issued, licensed, and/or received, and no royalties have been received from this research. The reviewer FS and handling editor declared their shared affiliation.
